# Comparative Plastome Analysis Between Endangered Mangrove Species *Acanthus ebracteatus* and *Acanthus* Relatives Provides Insights into Its Origin and Adaptive Evolution

**DOI:** 10.1002/ece3.70566

**Published:** 2024-11-20

**Authors:** Zanshan Fang, Danfeng Li, Haien Murong, Meng He, Yuqi Liu, Jiaxuan Liu, Jiaxiao Wu, Yingqi Li, Yongyu Li, Xiang Jin, Yuchen Yang, Ying Zhang

**Affiliations:** ^1^ Hainan Mangrove Research Institute Hainan Academy of Forestry Haikou China; ^2^ Mangrove Institute, Research Center of Integrated Protection and Utilization of Mangrove Rare and Endangered Species, Zhanjiang Key Laboratory of Mangrove Ecosystem Protection and Restoration Lingnan Normal University Zhanjiang China; ^3^ Ministry of Education Key Laboratory for Ecology of Tropical Islands, Key Laboratory of Tropical Animal and Plant Ecology of Hainan Province, College of Life Sciences Hainan Normal University Haikou China; ^4^ State Key Laboratory of Biocontrol, School of Ecology Sun Yat‐sen University Shenzhen China

**Keywords:** adaptive evolution, divergence time, endangered mangrove, molecular markers, plastome

## Abstract

*Acanthus ebracteatus* is a typical true mangrove species with great ecological and medicinal values. However, it has become endangered in China. Moreover, because of the similar morphology and distribution, it is commonly confused with the congeneric mangrove species, *A. ilicifolius*, which poses challenges to the protection and proper medicinal utilization of *A. ebracteatus*. Plastomes provide a solution for molecular identification and adaptive evolution investigation of plants. In this study, we dissected the complete plastome for *A. ebracteatus* and performed comparative analysis to *A. ilicifolius* and three non‐mangrove relatives ( *A. montanus* , *A. leucostachyus* and *A. mollis* ). Both plastome sequences and structure are highly conserved between the two mangrove species, while less similar between mangrove and non‐mangrove species. Phylogenetic analysis suggested that the mangrove species were divergent from the non‐mangrove groups at approximately 15.15 million years ago (Mya), where early to middle Miocene global warming and high sea level might act as one of the main forces driving the mangrove lineage entering into intertidal environments. Furthermore, 12 single nucleotide polymorphisms (SNPs) and 10 insertions/deletions (indels) were detected between the plastomes of *A. ebracteatus* and *A. ilicifolius*. PCR validation further demonstrated the effectiveness of the plastid marker in distinguishing the two sibling mangrove species. Taken together, our study broadens the understanding of the origin and evolution of *Acanthus* mangrove plants, and provided valuable information on the correct identification and protection of endangered mangrove species *A. ebracteatus* .

## Abstract 2

In this study, we assembled and characterized the complete cp of five *Acanthus* species, and showed a higher similarity in cp genome sequences and structures between two mangrove species than their terrestrial relatives, mirroring a monophyletic origin of mangrove plants in this genus and a very recent divergence between the two mangrove species. We further identified some variants and indels in cp genomes, which can serve as informative markers for the two close‐related mangrove species. These new findings do not only offer reference data for *Acanthus* species, but also provide a better understanding of the origin and evolution of the mangrove species of *Acanthus* genus.

## 1 Introduction

*Acanthus* is the unique genus consisting of both true mangrove and non‐mangrove species (Tomlinson 1986). In this genus, two true mangrove species, *Acanthus ilicifolius* and *A. ebracteatus*, and one non‐mangrove species *Acanthus leucostachyus* are naturally distributed in China (Wang and Wang 2007). Like other mangrove plants, both *A. ilicifolius* and *A. ebracteatus* play important roles in supporting the sustainability of intertidal ecosystems (Tomlinson 1986). Additionally, *A. ebracteatus* has several medicinal usages in traditional Chinese medicine, especially in treating rheumatism, cough, snake‐bite, chronic fever, asthma, hepatitis, intestinal worms, herpes zoster, leucorrhea, menstrual disorders, rash and some other skin diseases (Somchaichana, Bunaprasert, and Patumraj 2012; Jia et al. 2022). A recent research paper highlighted its potential application values in producing nutraceuticals and cosmeceuticals (Olatunji et al. 2022). Despite its ecological and medicinal importance, *A. ebracteatus* has become endangered in China. The field survey in 2022 revealed that, the biggest natural population of *A. ebracteatus* in China is in the Leizhou Peninsula and its total distribution area is approximately 9000 m^2^ (Lin et al. 2022). Comparatively, only 1831 *A. ebracteatus* plants were recorded in two small patches in Guangxi with a total distribution area of ~800 m^2^ (Huang et al. 2020). Now *A. ebracteatus* has been listed in the China Red List of Biodiversity (higher plants volume) and it is in urgent need of protection (Lin et al. 2022; Zhang et al. 2021). In recent years, cutting has been successfully applied to breed *A. ebracteatus* in laboratories (Zhang et al. 2023). However, our poor knowledge on the adaptive mechanisms of *A. ebracteatus* to environmental stresses largely restricts the afforestation and restoration of *A. ebracteatus* under natural conditions. In contrast to *A. ebracteatus*, its sibling species *A. ilicifolius* is widely distributed, and in some areas, it has been reported to cover more than 90% of the lower layer of the mangrove forests (Tang et al. 2023). *A. ebracteatus* has high morphological similarity with *A. ilicifolius*, and the main difference is their flower color, which makes these two species difficult to be distinguished in the wild (Tomlinson 1986). And these two closely‐related species are often used without the correct distinction, which brings more difficulty to the protection, afforestation and medication utilization of *A. ebracteatus*.

With the development of high‐throughput sequencing technologies, genomic data have been widely applied in molecular identification and adaptive analysis in plant species. He et al. (2022) performed a comprehensive investigation into the origin and evolution of mangroves using large‐scale genome sequencing data. However, no whole genome sequence is available to either *A. ilicifolius* or *A. ebracteatus*, because both species are polyploid, and the complex genome structures pose great challenges to genome assembly (Wang et al. 1998; Wang et al. 2023). Compared to the nuclear genome, plastid/chloroplast DNA has a smaller size, a modest synonymous substitution rate and no sexual recombination (Wolfe et al. 1987; Salih et al. 2017). These advantages make the plastome (hereafter, we use plastome and chloroplast genome interchangeably) an ideal system for phylogeny investigation and divergence time estimation among different plant lineages (Nizam et al. 2022; Temel et al. 2022; Wei and Li 2022). Moreover, plastomes also offer a valuable genetic resource for developing molecular markers for distinguishing closely related species (He et al. 2024).

Recent studies have largely enriched plastome resources of mangrove plants, and revealed that plastome variations between mangrove and non‐mangrove species of the same family may contribute to adaptation to different stress factors, such as high salinity, temperature, and ultraviolet (UV) radiation (Asaf et al. 2021; Ruang‐Areerate et al. 2021; Tan et al. 2023; Temel et al. 2022; Xu et al. 2022; Zhang et al. 2020). For Acanthaceae species, plastid DNA has been widely utilized for exploring their phylogenetic relationships (Alzahrani et al. 2020; Temel et al. 2022; Wei and Li 2022). We previously conducted a phylogenetic analysis using nrITS and three plastid DNA markers and revealed that *A. ilicifolius* and *A. ebracteatus* form a separate clade from the non‐mangrove species, indicative of the monophyletic origin of the two *Acanthus* mangrove species (Yang et al. 2015). However, given the relatively low substitution rate of plastid DNA, that study cannot provide enough resolution to investigate the genetic divergence between the two closely related species *A. ebracteatus* and *A. ilicifolius*, which leaves a knowledge gap on the differentiation and evolutionary history of the *Acanthus* mangrove species.

In this study, we de novo sequenced and dissected the complete plastome for *A. ebracteatus* and performed a comparison to other *Acanthus* species to explore: (1) global structural variation among different *Acanthus* plastomes; (2) the phylogenetic relationship among *Acanthus* species and the divergence times of two *Acanthus* mangrove species with terrestrial non‐mangrove relatives; and (3) the most variable regions in plastome that can be used for developing DNA markers for distinguishing two *Acanthus* mangrove species. These findings unravel the origin and evolution of *Acanthus* mangrove plants, and provide effective genetic markers for distinguishing *A. ebracteatus* from *A. ilicifolius*, which can broaden our knowledge on environmental adaptation of *A. ebracteatus* and improve its correct recognition and protection in the field.

## 2 Materials and Methods

### 2.1 Sample Preparation, Sequencing, Assembly, and Annotation

The sequenced *Acanthus* mangrove species (*A. ilicifolius* and *A. ebracteatus*) were collected in Zhanjiang Mangrove National Nature Reserve, Guangdong, China. The three non‐mangrove relatives, *A. leucostachyus*, *A. montanus*, and *A. mollis*, were collected in Xishuangbanna Tropical Botany Garden, Yunnan, China, South China National Botanical Garden, Guangdong, China, and Shanghai Chenshan Botanical Garden, Shanghai, China, respectively. Ten healthy leaves were collected from each plant, immediately frozen in liquid nitrogen in the field, and stored in a −80°C freezer. All the voucher specimens were numbered and deposited in Lingnan Normal University (A. ilicifolius: Ai‐001; *A. ebracteatus*: Ae‐001; *A. leucostachyus*: Al‐001; *A. montanus*: Amon‐001; and *A. mollis*: Amol‐001).

Genomic DNA was extracted from frozen leaves using the DP350 Kit (TIANGEN BIOTECH [Beijing] Co. Ltd., Beijing, China). The sequencing library was constructed for each sample using the NEB Next Ultra DNA Library Prep Kit for Illumina (New England Biolabs, Ipswich, MA, USA) following the instructions of the manufacturer. The sequencing was carried out in an Illumina HiSeq X‐ten platform (Illumina Inc. San Diego, CA, USA). The quality of the sequencing reads was assessed by FastQC (https://www.bioinformatics.babraham.ac.uk/projects/fastqc/) and reads of low quality were trimmed out using Fastp v.0.11.0 (Brown, Pirrung, and McCue 2017). The clean reads of each *Acanthus* species were then assembled by GetOrganelle v.1.7.5.0 (Jin et al. 2020), using the published plastome of *A. ilicifolius* (NCBI accession number: MW174172) as reference. Gene annotation was conducted using GeSeq (Tillich et al. 2017), and the annotation results for coding sequences (CDSs) were manually confirmed with the BLAST program and those for tRNA‐coding genes were checked by tRNAscan software (Chan and Lowe 2019). Genes that have been reported as pseudogenes in previous studies were picked out and their putative protein products were manually examined. The genes were defined as “pseudogenes” if their products contain any premature termination codons (PTCs) and the PTCs cause a large fragment loss. The structure of each plastome was visualized in Chloroplot (Zheng et al. 2020).

### 2.2 Plastome Structure Comparison Among *Acanthus* Species

Comparative analyses were conducted to investigate the structural variations among five *Acanthus* plastomes. In particular, the nucleotide composition of each plastome was characterized using Bioedit software (https://bioedit.software.informer.com/). The boundaries at the junctions of inverted repeat (IR) and two single‐copy (SC) regions were identified using the online tool Irscope (https://irscope.shinyapps.io/irapp/), and the prediction results were checked and visualized manually. Sequence divergences among five *Acanthus* plastomes were obtained and visualized using the mVISTA program with the Shuffle‐LAGAN algorithm (Frazer et al. 2004) and *A. ilicifolius* set as the reference. Nucleotide diversity (π) was computed for both gene body and intergenic spacer regions across the five aligned *Acanthus* plastomes using DnaSP v6 software (Rozas et al. 2017) to investigate the hypervariable regions in *Acanthus* plastomes. To develop molecular specific markers for distinguishing the two mangrove species (*A. ilicifolius* and *A. ebracteatus*), *A. ilicifolius* plastome was aligned to that of *A. ebracteatus* using the mummer program of the MUMmer software (Marcais et al. 2018), and only the unique matches between the two species were retained for the subsequent analysis. The correspondence in the sequence coordinates and orientation between the two plastomes were determined by the nucmer utility, and all the single nucleotide polymorphisms (SNPs) and insertions/deletions (indels) were identified by the show‐snps program of the MUMmer software (Marcais et al. 2018).

### 2.3 Gene Selection Pressure Analysis

A total of 79 homologous protein‐coding genes were used to investigate selection pressures in two *Acanthus* mangrove species versus their terrestrial non‐mangrove relatives in Acanthaceae (*A. leucostachyus*, *A. montanus*, *A. mollos*, *Andrographis paniculata*, *Blepharis ciliaris* and *Barleria prionitis*). For each species pair, genes were first aligned, and the numbers of non‐synonymous (Ka) and synonymous (Ks) nucleotide substitutions and their ratio (Ka/Ks) were calculated using Ka/Ks Calculator v 2.0 (Wang et al. 2010). The Ka/Ks ratios were then visualized using the heatmap function in R.

### 2.4 Codon Usage Bias and Aversion Index Analysis

Codon usage bias (CUB) is a key factor influencing gene expression and protein translation, and can reflect the evolutionary history and relationships among different species (Parvathy et al. 2022). Fifty‐one protein‐coding genes with a size of CDS > 300 bp were selected for inferring synonymous codon usage and aversion indices in *Acanthus* plastomes. The corresponding indices such as the effective number of codons (ENC), codon bias index (CBI), and relative synonymous codon usage (RSCU) were computed using DnaSP v6 (Rozas et al. 2017). The optimal codons were calculated based on the △RSCU method (Han et al. 2022), and the codon aversion motifs, which refer to a collection of codons not present in genes, were identified for the plastome of each *Acanthus* species using the CAM approach (Miller et al. 2019).

### 2.5 Polymerase Chain Reaction (PCR) Amplification and Gene Sequence Validation

To validate the highly divergent regions identified in *Acanthus* plastomes, two protein‐coding genes (*psbK* and *ycf1*) and two intergenic spacer regions with specific DNA deletion in *A. mollis* (*ndhF* and *ndhA*) were selected for PCR amplification and sequencing for five individuals of each *Acanthus* species. For each species, the PCR procedure was carried out on five individuals as biological replicates. Each 20 μL PCR reaction contained 1 μg genomic DNA, 1 μL of each primer, and 17 μL Taq DNA polymerase mix (TaKaRa Bio. Inc. Kusatsu, Shiga, Japan). The sequences, annealing temperature, and concentration of each pair of PCR primers were listed in Table S1.

To distinguish the two sibling mangrove species, *A. ebracteatus* and *A. ilicifolius*, we selected the intergenic spacer region *atpB* *‐* *rbcL* with an insertion in *A. ebracteatus* as candidate marker. Universal PCR primers were designed based on the flanking conserved sequences of the two species (see Table S1 for detailed primer sequences and characters). To verify the efficiency and reliability of this marker, 10 individuals of each species were collected from natural populations, and the target locus was amplified and sequenced using the universal primers we designed. PCR procedures were implemented using the same reaction system described above.

### 2.6 Phylogenetic Analysis and Divergence Times Evaluation

To infer the phylogenetic relationship among *Acanthus* species, a phylogenetic analysis was performed for 13 Acanthaceae plastomes, with four species from Phrymaceae, Gesneriaceae, Scrophulariaceae, and Bignoniaceae as outgroups (their accession numbers were listed in Table S2). The plastome sequences were aligned using MAFFT software (Katoh et al. 2019) with default parameter settings. A phylogenetic tree was constructed by a maximum likelihood (ML) approach in RAxML‐HPC v.8.2.12 (Stamatakis 2014), where the GTR + GAMMA + I substitution model was selected and the bootstrap replicate was set to 1000.

The divergence time among *Acanthus* species was estimated using BEAST 2 v.2.1.3 (Bouckaert et al. 2019). The input files for BEAST 2 analysis were generated using the BEAUti interface (Drummond et al. 2012), according to the alignment result of MAFFT. The topology of the tree prior of the 17 species was set based on their phylogenetic relationships inferred by the ML analysis. Calibrated Yule Model tree prior and strict clock model were used, and Gamma Category Count and iterations of Markov chain Monte Carlo (MCMC) were set as 4 and 1,000,000, respectively. Two calibration priors were set to the stem nodes of the most recent common ancestor (MRCA) of all the analyzed Acanthaceae species (offset: 79 million years ago (Mya); sigma: 0.5) and Acantheae species (offset: 44 Mya; sigma: 0.5), respectively, based on the previously reported divergence time between Acantheae and Ruellieae (the mean was approximately 79 Mya and the 95% credibility interval (CI) ranged from 65 to 102 Mya) and the divergence time of crown clades of Acantheae (44 Mya with a 95% CI of 42 to 54 Mya) (Tripp et al. 2013). In addition, the fossil record indicated that Barlerieae taxa emerged at ~5.3–23.8 Mya (Tripp et al. 2013; Tripp and Tsai 2017), thus a prior (offset: 5.5 Mya; sigma: 0.1) was placed on the stem node of Barleria prionitis and Andrographis paniculata. The trees generated by BEAST 2 were summarized onto a maximum clade credibility tree using TreeAnnotator v.2.7.4.

## 3 Results

### 3.1 Features of Plastome Organization and Structure

**FIGURE 1:**
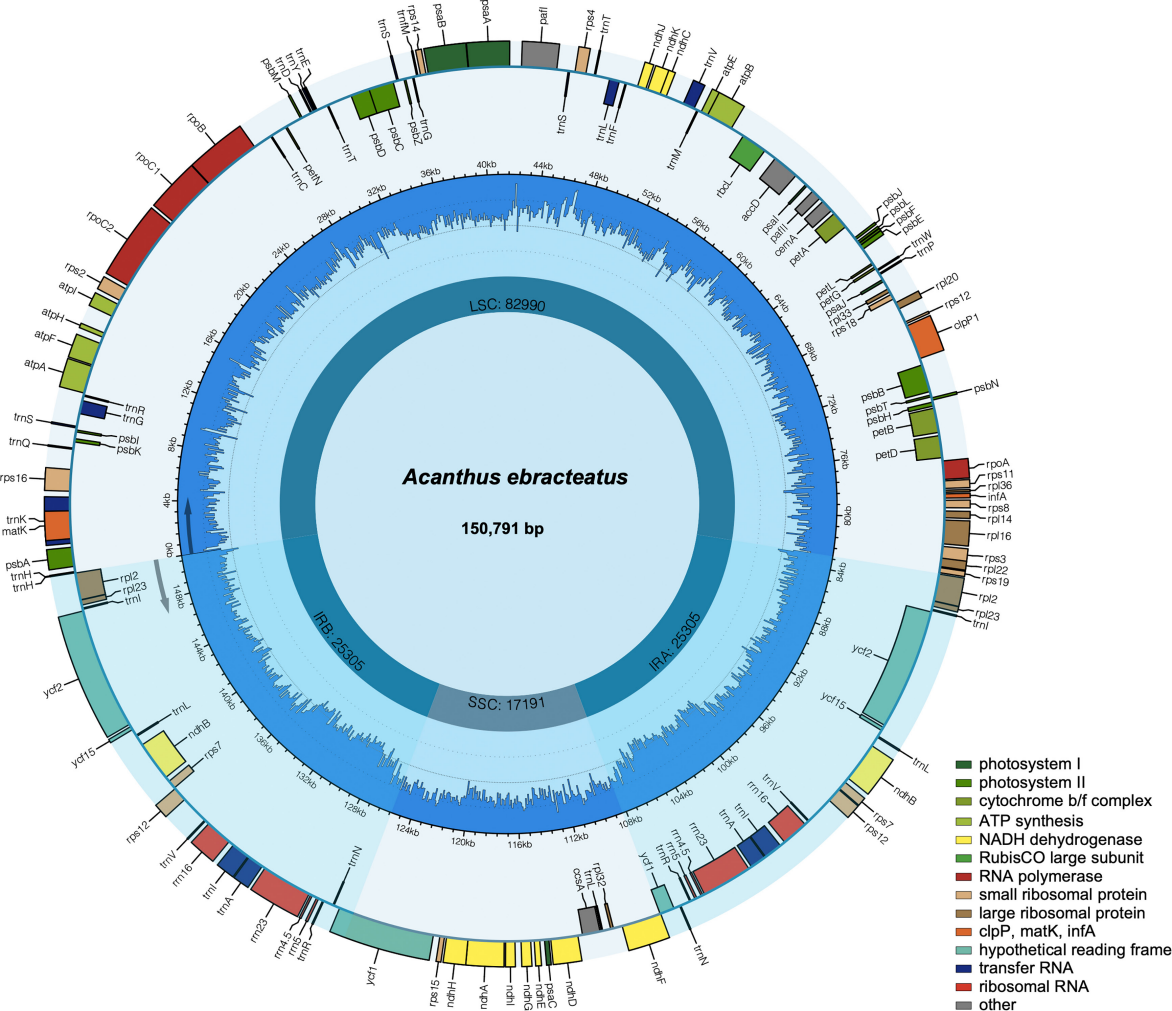
Plastome map of *Acanthus ebracteatus*. Genes transcribed clockwise and counterclockwise are listed inside and outside of the circle, respectively, where the direction is presented by arrows. Genes are color‐coded by their functional classification. The GC content of the genome is depicted as the proportion of the shaded parts of each section.

The size of five complete plastomes of *Acanthus* ranged from 150,337 bp (*A. mollis*) to 150,791 bp (*A. ebracteatus*) with typical quadripartite structures, containing a large single‐copy (LSC) region (82,612–82,990 bp) and a small single‐copy (SSC) region (17,019–17,191 bp) separated by two IR regions (25,081–25,353 bp) (Figure 1, Figure S1 and Table 1). Among the five *Acanthus* species, two true mangrove species, *A. ilicifolius* and *A. ebracteoctus* have the longest plastome and LSC region. All five plastomes were extremely conserved in GC contents and gene numbers, which presented 133 genes, including 88 protein‐coding genes, eight rRNAs, and 37 tRNA‐coding genes. Furthermore, all five species contained one pseudogene, except *A. mollis*, which had three pseudogenes (*ycf1*, *ndhA* and *ndhF*). Two introns were detected in each of the two protein‐coding genes, *clpP1* and *pafI*, while a single intron was respectively detected in each of nine protein‐coding genes (*atpF*, *ndhA* [except in *A. mollis*], *ndhB*, *petB*, *petD*, *rpl16*, *rpl2*, *rpoC1* and *rps16*) and six tRNA genes (*trnA‐UGC*, *trnG‐UCC*, *trnI‐GAU*, *trnK‐UUU*, *trnL‐UAA* and *trnV‐UAC*) (Table 2). Among the 88 protein‐coding genes, 85 used the standard start codon AUG, while two genes (*rps19* and *psbC*) initiated with the alternative start codon GUG, and the one ndhD started with the codon ACG.

**TABLE 1:** Comparative analyses on the basic features of the plastomes of five *Acanthus* species.

	*Acanthus ilicifolius*	*Acanthus ebracteatus*	*Acanthus montanus*	*Acanthus leucostachyus*	*Acanthus mollis*
Length (bp)	150,723	150,791	150,569	150,569	150,337
GC content (%)	38.42	38.4	38.26	38.26	38.12
LSC length (bp)	82,928	82,990	82,797	82,797	82,612
SSC length (bp)	17,191	17,191	17,610	17,262	17,019
IR length (bp)	25,302	25,302	25,081	25,255	25,353
Gene number	133	133	133	133	133
Gene number in IR regions	38	38	38	38	38
Protein‐coding gene number	88	88	88	88	88
Pseudogene number	1	1	1	1	3
rRNA gene number	8	8	8	8	8
tRNA gene number	37	37	37	37	37
Intron‐containing gene number	17	17	17	17	16

**TABLE 2:** Genes in the plastomes of five *Acanthus* species.

Category	Gene group	Gene name
Photosynthesis	Photosystem I	*psaA, psaB, psaC, psaI, psaJ*
	Photosystem II	*psbA, psbB, psbC, psbD, psbE, psbF, psbH, psbI, psbJ, psbK, psbL, psbM, psbN, psbT, psbZ*
	Cytochrome b/f complex	*petA, petB, petD, petG, petL, petN*
	ATP synthesis	*atpA, atpB, atpE, atpF, atpH, atpI*
	Large subunit of RuBisCo	*rbcL*
	NADH dehydrogenase	*ndhA* ^b^, *ndhB, ndhC, ndhD, ndhE, ndhF, ndhG, ndhH* ^b^ *, ndhI, ndhJ, ndhK*
	Ribosomal RNA genes	*rrn4.5, rrn5, rrn16, rrn23*
	Small subunit ribosomal RNA genes (SSU)	*rps2, rps3, rps4, rps7, rps8, rps11, rps12, rps14, rps15, rps16, rps18, rps19*
	Large subunit ribosomal RNA genes (LSU)	*rpl2, rpl14, rpl16, rpl20, rpl22, rpl23, rpl32, rpl33, rpl36*
Self‐replication	RNA polymerase	*rpoA, rpoB, rpoC1, rpoC2*
	Transfer RNA genes	*trnA‐UGC, trnC‐GCA, trnD‐GUC, trnE‐UUC, trnF‐GAA, trnfM‐CAU, trnG‐GCC, trnG‐UCC, trnH‐GUG, trnI‐CAU, trnI‐GAU, trnK‐UUU, trnL‐CAA, trnL‐UAA, trnL‐UAG, trnM‐CAU, trnN‐GUU, trnP‐UGG, trnQ‐UUG, trnR‐ACG, trnR‐UCU, trnS‐GCU, trnS‐GGA, trnS‐UGA, trnT‐GGU, trnT‐UGU, trnV‐GAC, trnV‐UAC, trnW‐CCA, trnY‐GUA*
	Maturase	*matK*
	Envelope membrane factor	*cemA*
Other genes	Subunit of acetyl‐CoA	*accD*
	C‐type cytochrome synthesis gene	*ccsA*
	Protease	*clpP*
	Translational initiation factor	*infA*
	Hypothetical chloroplast reading	*ycf1* ^a^ *, ycf2, pafI, pafII, yfc15*

^a^ Pseudogenes in all five *Acanthus* species.

^b^ Pseudogenes only in *Acanthus mollis*.

### 3.2 Comparative Structural Analysis of Five *Acanthus* Plastomes

**FIGURE 2:**
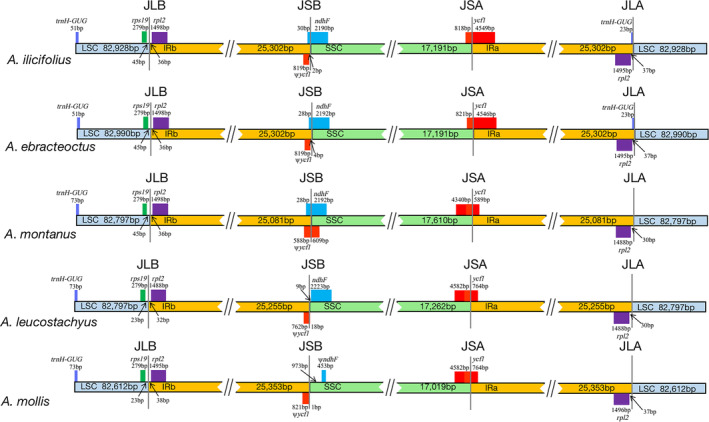
Comparison of LSC, IRs, and SSC junction positions among five *Acanthus* plastomes.

The five *Acanthus* plastomes exhibited obviously differences in the IR‐SC boundary regions (Figure 2), especially between mangrove and non‐mangrove species. For instance, in two mangrove species, a pseudogene fragment *ψycf1* was found in IRb region that was 2 and 4 bp away from the IRb‐SSC boundary, and gene *ndhF* was mainly in the SSC region with 30 and 28 bp spanned across the boundary, leading to 28 and 24 bp overlaps with *ψycf1*. Comparatively, *ψycf1* of *A. montanus* was ~46%–61% longer than in those in the other four species, and it was 637 bp overlapped with *ndhF*. In *A. leucostachyus*, gene *ndhF* was entirely located in SSC region, and 27 bp away from *ψycf1*. It is noteworthy that, in *A. mollis*, *ndhF* specialized to a 453 bp pseudogene fragment (*ψndhF*) in the SSC region, and the distance between *ψycf1* and *ψndhF* was 974 bp. At the SSC‐IRa boundary, the majority of *ycf1* sequences in two mangrove species were located in the IRa region, while those of the three non‐mangrove species were mainly in the SSC region. Furthermore, at the junction of IRa and LSC regions, gene *trnH‐GUG* of two mangrove species spanned the junction of IRa and LSC regions, with 23 bp in the IRa region, while it was specifically located in the LSC region in the three non‐mangrove species.

**FIGURE 3:**
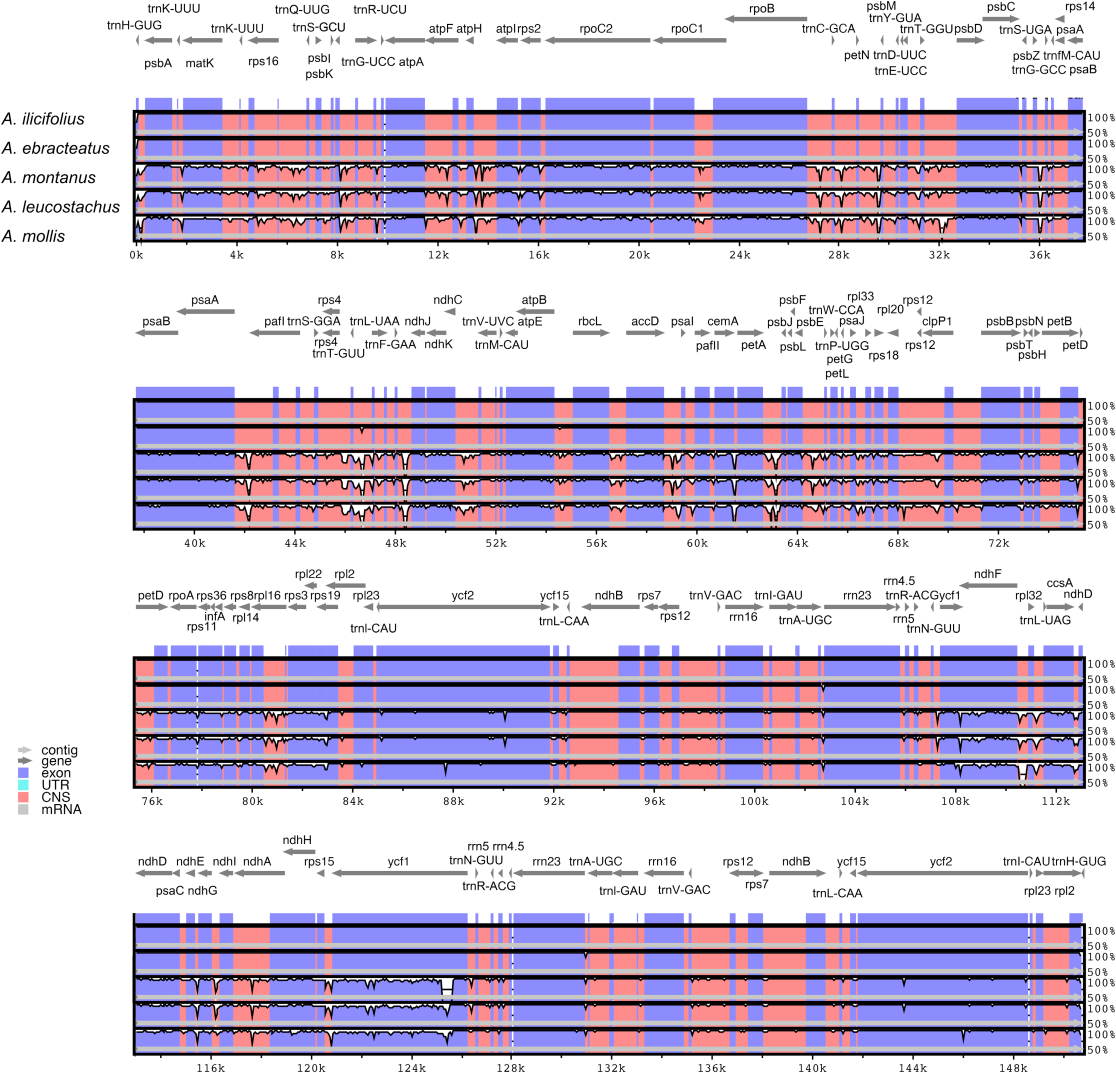
Sequence similarities among the five *Acanthus* plastomes by mVISTA, with the plastome of *Acanthus ilicilolius* as the reference.

**FIGURE 4:**
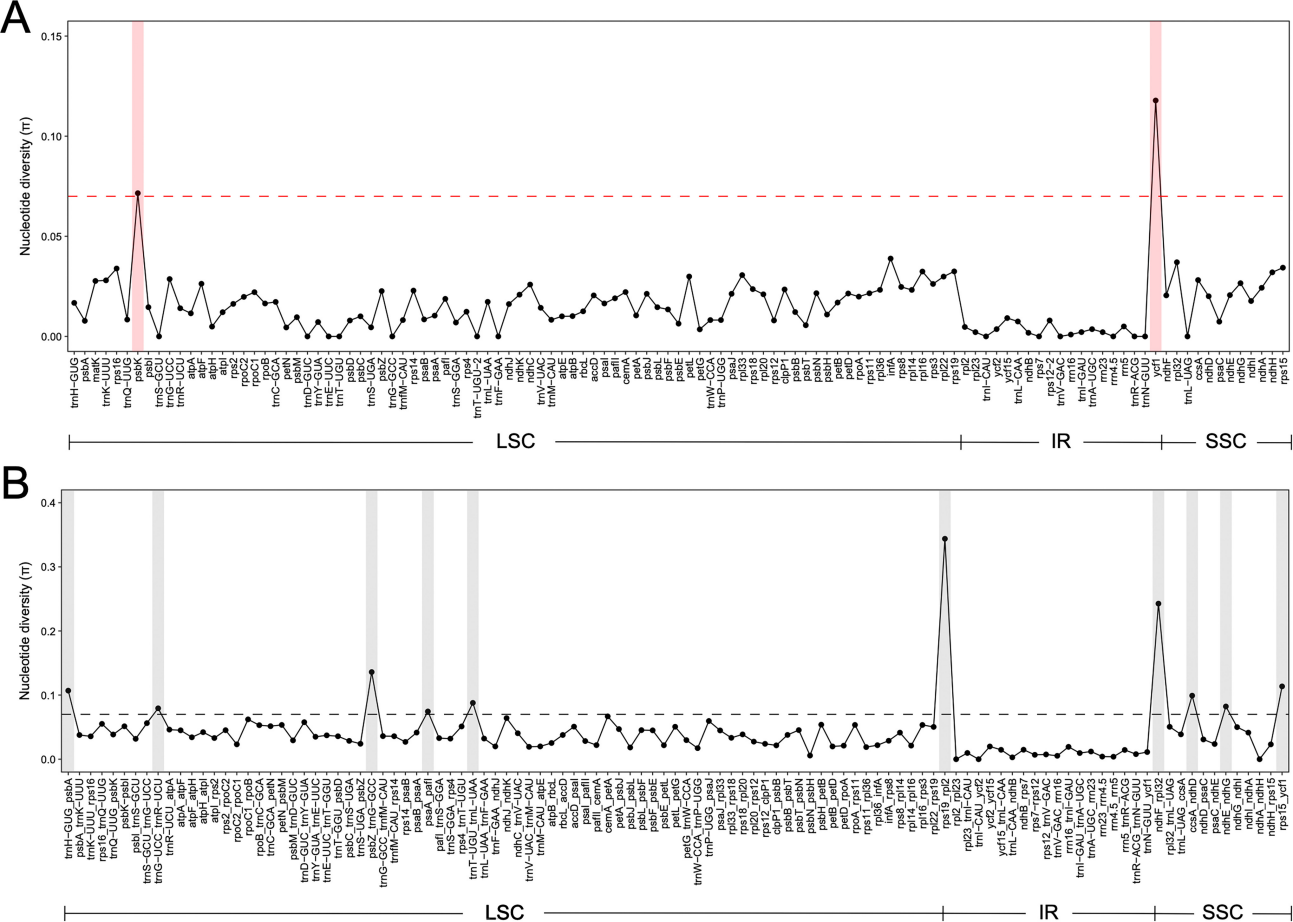
Nucleotide diversity (π) of plastid loci across five *Acanthus* species. (A) Distribution of π values for genes. (B) Distribution of π values for intergenic spacer regions. The dash lines illustrate a threshold of *π* = 0.070.

The sequence divergence among the five *Acanthus* plastomes is illustrated in Figure 3. Two *Acanthus* mangrove plastome sequences were almost identical, while they were less conserved between mangrove and non‐mangrove species (Figure 3). A total of 12 plastid loci were found to display a high level of sequence variations among the five species, with nucleotide diversity (π) ranging from 0.072 to 0.344, including two protein‐coding genes (*psbK* and *ycf1*) and 10 intergenic spacer regions (*trnH‐GUG_psbA*, *trnG‐UCC_trnR‐UCU*, *psbZ_trnG‐GCC*, *psaA_pafI*, *trnT‐UGU_trnL‐UAA*, *rps19_rpl2*, *ndhF_rpl32*, *ccsA_ndhD*, *ndhE_ndhG* and *rps15_ycf1*) (Figure 4 and Table 3). The high divergence of sequence among *Acanthus* species was verified by PCR validation (Figure S2). Comparatively, the largest variations occurred between mangrove and non‐mangrove species, which was consistent with the results of comparative plastome analysis. For instance, in *psbK*, a DNA fragment deletion of TGTATT and an insertion of TATTCG were specifically detected in two mangrove species, but not in three non‐mangrove relatives (Figure S2).

**TABLE 3:** Highly variable regions in *Acanthus* plastomes.

Region	Nucleotide diversity (π)	Number of variants	Region length
*psbK*	0.072	22	186
*ycf1*	0.118	203	819
*trnH‐GUG_psbA*	0.107	55	287
*trnG‐UCC_trnR‐UCU*	0.079	29	257
*psbZ_trnG‐GCC*	0.136	45	384
*psaA_pafI*	0.074	79	650
*trnT‐UGU_trnL‐UAA*	0.088	98	802
*rps19_rpl2*	0.344	25	82
*ndhF_rpl32*	0.243	193	478
*ccsA_ndhD*	0.099	39	257
*ndhE_ndhG*	0.082	24	192
*rps15_ycf1*	0.114	69	362

### 3.3 Sequence Variation Between *A. ilicifolius* and *A. ebracteatus* and Marker Development

**FIGURE 5:**
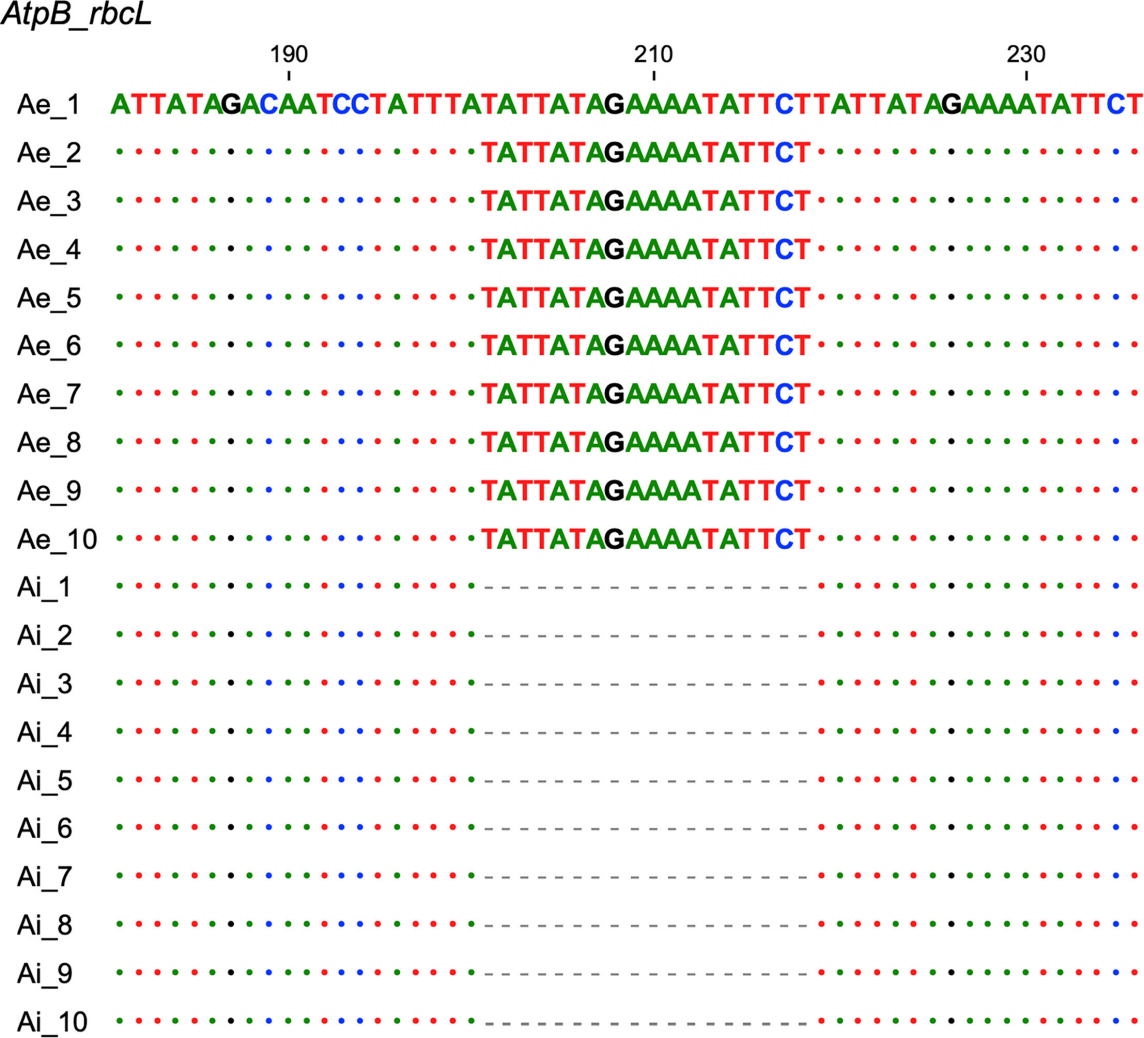
Sequence alignment of the indel in the fragment of *atpB_rbcL*, which can be used for distinguishing *A. ebracteoctus* from *A. ilicifolius*. The numbers above the alignment represent the nucleotide positions in sequencing products. Ten individuals of each species were collected for the validation. Ae, *A. ebracteoctus*; Ai, *A. ilicifolius*.

Using the *A. ilicifolius* plastome as reference, we detected the SNPs and indels in the plastome of *A. ebracteatus*. Between the two mangrove species, three and nine SNPs were detected in protein‐coding genes and intergenic regions, respectively (Table S3). In addition, there were eight insertions and two deletions detected in *A. ebracteatus* plastome compared to *A. ilicifolius*. These identified SNPs and indels might be used as molecular markers for distinguishing the two *Acanthus* mangrove species. For instance, compared to *A. ilicifolius*, *atpB‐rbcL* contained one insertion of 18 bp in *A. ilicifolius*. PCR validations on 10 individuals of each species showed a high intra‐species consistency in sequence, while an apparent difference between different species (Figure 5), demonstrating the effectiveness of using this locus to distinguish the two sibling species.

### 3.4 Genes Under Positive Selection

**FIGURE 6:**
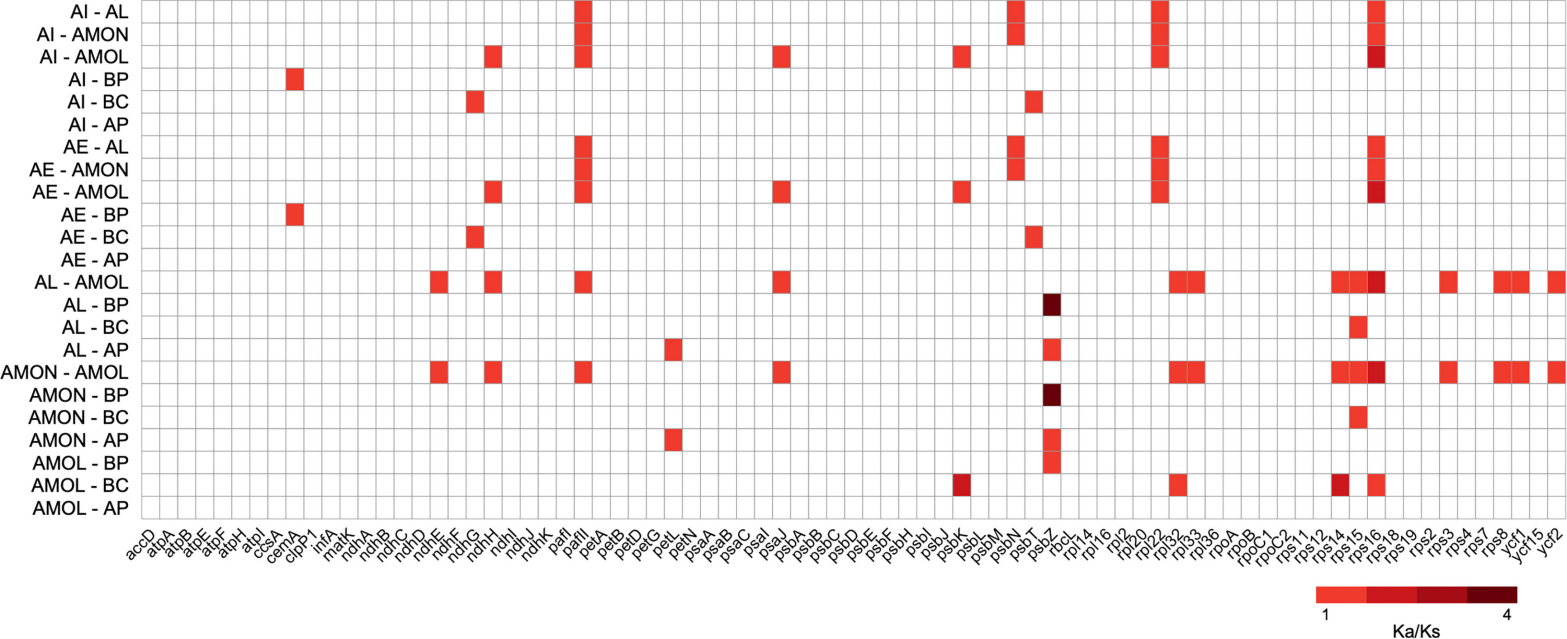
Heatmap of Ka/Ks ratios between every compared species in 79 plastid protein‐coding gene. The scale ratios associated with each value are shown in the key beside the figure. AI, *A. ilicifolius*; AE, *A. ebracteoctus*; AL, *A. leucostachyus* ; AMON, *A. montanus* ; AMOL, *A. mollis* ; BP, *Barleria prionitis* ; BC, *Blepharis ciliaris*; AP, *Aphelandra knappia*.

To identify putative genes under positive selection, the Ka/Ks ratios were computed for 79 conserved plastid protein‐coding genes between *Acanthus* mangrove species and their non‐mangrove relatives of Acanthaceae (Figure 6). Most Ka/Ks ratios were lower than one. In contrast, the Ka/Ks ratios of nine genes, *ndhG*, *ndhH*, *pafII*, *psaJ*, *psbK*, *psbN*, *psbT*, *rpl22* and *rps16*, were greater than one in some certain species pairs, indicative of putative positive selection during their evolution. In particular, the average Ka/Ks ratios of genes *psbN* and *rpl22* were 1.261 and 1.221, respectively, between two *Acanthus* mangrove plants and non‐mangrove relatives *A. leucostachyus* and *A. montanus*, and when regarding the two mangrove species and the non‐mangrove plant *A. mollis*, the five genes *psbK*, *ndhH*, *pafII*, *psaJ* and *rps16* were identified with average Ka/Ks ratios ranging from 1.059 to 1.707, indicating they may be under positive selection.

### 3.5 Patterns of Codon Usage and Aversion

In the five *Acanthus* plastomes, 88,125–91,385 bp of CDS consisted of 18,885–20,007 codons. Leucine (L) was the most abundant amino acid in all five species, whereas cysteine (C) was the least with regardless of the stop codons (Table S4). The most commonly used codon was AUU with a number of 837 in both two mangrove plastomes, while the least used codon was UGC (54 in both two species). All the 20 examined amino acids, except methionine and tryptophan, displayed an apparent usage bias among different synonymous codons (Figure S3 and Table S4). A total of 30 codons were identified with an RSCU > 1, indicative of a more frequent usage than others. Of them, AGA for arginine showed the highest RSCU values (1.838–1.88) in all five species. Moreover, the third base of these codons was primarily A or U, while those with RSCU value < 1 mainly ended with G or C. A similar pattern was also observed in other *Acanthus* and Acanthaceae plants (Alzahrani et al. 2020; Yaradua et al. 2019).

The patterns of codon usage and aversion were explored and compared among the five *Acanthus* plastomes. The gene with the top and bottom 10% of ENC values in each species was selected and compared among these five species to evaluate varying levels of CUB (Table 4). Corresponding to the similar structure of plastomes, two *Acanthus* mangrove species displayed high similarities in ENC values for both low and high groups. Furthermore, a high consistency in ENC values was also observed between two non‐mangrove species: *A. montanus* and *A. leucostachyus*. Comparatively, *A. mollis* displayed apparent specificity in CUB, for example, ndhH (54.76) and pafI (55.06) were of high ENC values only in *A. mollis*.

**TABLE 4:** The top and bottom 10% of effective number of codons (ENC) values of 51 CDSs among five *Acanthus* plastomes.

	*Acanthus* *ilicifolius*		*Acanthus* *ebracteatus*		*Acanthus* *montanus*		*Acanthus* *leucostachyus*		*Acanthus* *mollis*	
	Gene	ENC	Gene	ENC	Gene	ENC	Gene	ENC	Gene	ENC
Low group	*rps14*	37.80	*rps14*	37.80	*rps14*	40.27	*rps14*	40.27	*rps14*	37.50
	*rps8*	38.37	*rps8*	38.37	*rps8*	41.56	*rps8*	41.56	*rps8*	40.73
	*ndhA*	41.09	*ndhA*	41.09	*ndhG*	38.59	*ndhG*	38.59	*ndhG*	38.44
	*ndhE*	41.63	*ndhE*	41.63	*cemA*	41.60	*cemA*	41.60	*ndhE*	38.70
	*ndhG*	41.85	*ndhG*	41.85	*ndhF*	42.69	*ndhF*	42.69	*ndhF*	37.59
High group	*ycf2*	53.42	*ycf2*	53.42	*rpoA*	53.91	*rpoA*	53.91	*ndhH*	54.76
	*rps4*	53.56	*rps4*	53.56	*rps4*	54.56	*rps4*	54.56	*pafI*	55.06
	*ndhJ*	54.77	*ndhJ*	54.77	*rpl2*	54.45	*rpl2*	54.45	*ndhJ*	53.84
	*rps2*	55.09	*rps2*	55.09	*rps2*	55.09	*rps2*	55.09	*rps4*	56.21
	*pafII*	56.99	*pafII*	56.99	*pafII*	55.43	*pafII*	55.43	*pafII*	56.73

A total of 11 optimal codons were identified in five *Acanthus* plastomes (Table 5). Two true mangrove species displayed the same features (UUG, CGU, GAU, UCU, ACU, CCU and UCC), while the codon optimality was less conserved among the three non‐mangrove relatives. UUG and CGU were commonly used by all five species. Notably, mangrove and non‐mangrove species had species‐specific optimal codons; UUC was the optimal codon exclusively for two mangrove species, while AGU, AAU, ACA and GUA were only identified as optimal codons for non‐mangrove *Acanthus* species.

**TABLE 5:** The optimal codons of the five *Acanthus* species.

Species	Optimal codons										
*Acanthus* *ilicifolius*	UUG	CGU	GAU	UCU	ACU	CCU	UCC				
*Acanthus* *ebracteatus*	UUG	CGU	GAU	UCU	ACU	CCU	UCC				
*Acanthus* *montanus*	UUG	CGU				CCU		AGU		ACA	
*Acanthus* *leucostachyus*	UUG	CGU	GAU		ACU						GUA
*Acanthus* *mollis*	UUG	CGU	GAU	UCU				AGU	AAU		

**FIGURE 7:**
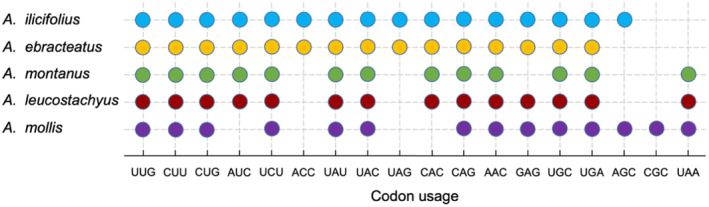
Codon aversion motifs of gene *rps11* in five *Acanthus* species. Circles represent used codons in each species, and different species were highlighted by different colors.

Codon aversion motifs have been used to investigate species phylogeny and evolution. Here, the CAM analysis on the CDSs of 51 protein‐coding genes revealed that only 11 genes (*atpB*, *ndhB*, *psaA*, *psbB*, *psbD*, *rpoA*, *rpoB*, *rpoC2*, *rps7*, *ycf1* and *ycf2*) showed the same codon aversion profiles across all the five *Acanthus* species. For all the genes except *atpA* and *rps11*, two mangrove species displayed the same codon aversion pattern (Table S5). For 18 genes, two mangrove species showed fewer unused codons than the non‐mangrove relatives. Eight codons were exclusively observed in the plastomes of mangrove species, including UGA and CGA in *accD*, AAG, CGG, and UAG in *rpoC1*, ACC in *rps11*, and UUG and AGG in *rps3* (Figure 7 and Table S5). The special codon aversion characteristics mirror a closer phylogenetic relationship between two *Acanthus* mangrove species than the non‐mangrove relatives.

### 3.6 Phylogenetic Relationships and Divergence Time Among Acantheae Species

**FIGURE 8:**
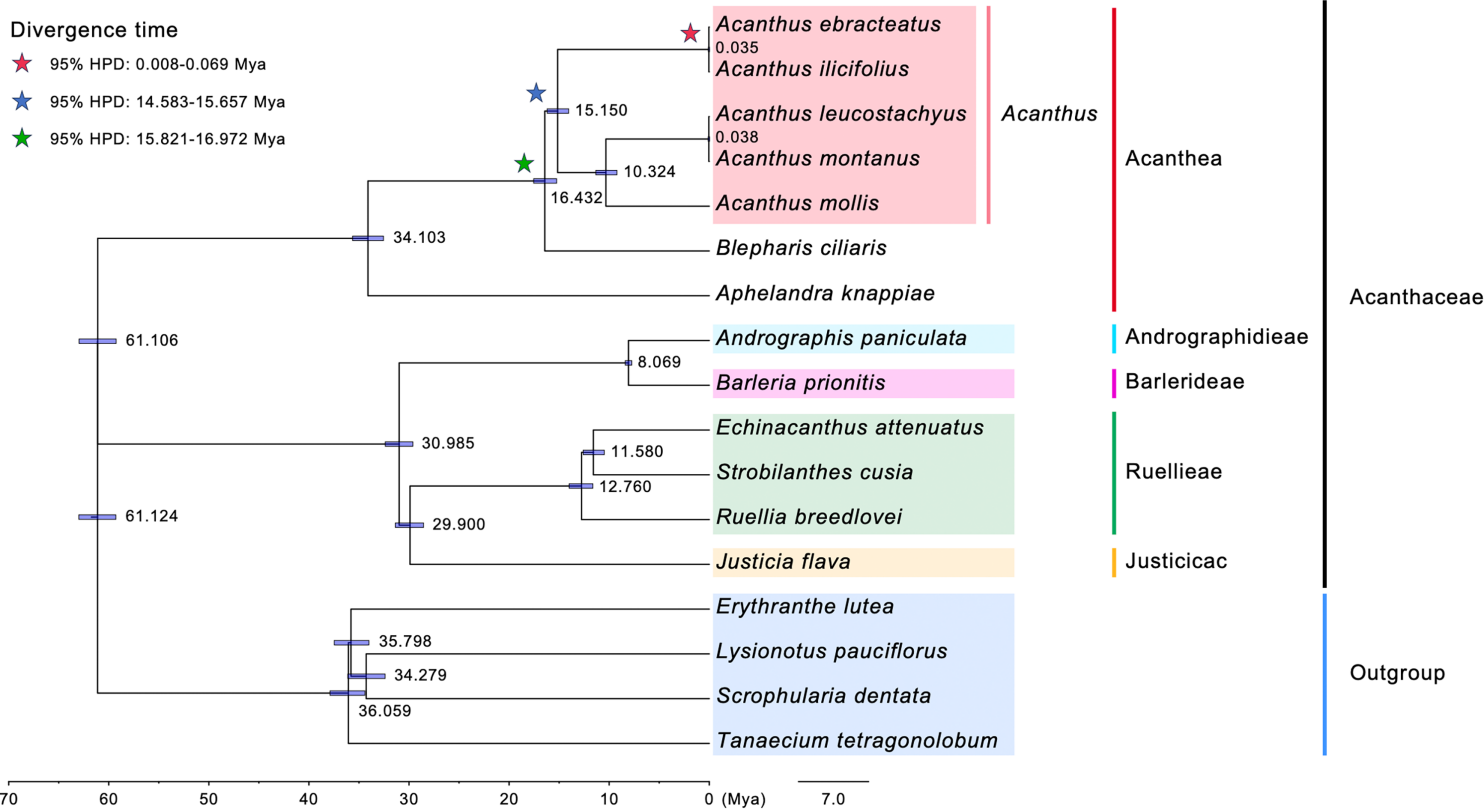
Phylogenetic analyses and divergence times estimation of Acantheae species based on the orthologous plastid coding sequences (CDS).

A phylogenetic tree was constructed for a total of 17 species to clarify the phylogenetic positions of the two *Acanthus* mangrove species in the genus. The 13 Acantheae species were clustered into two major clades with strong support: the Acantheae species, including those from *Acanthus*, *Blepharis*, and *Aphelandra*, formed one clade, while those from Andrographideae, Barlerieae, Ruellieae, and Justicieae belonged to the other clade (Figure S4), which was consistent with our prior knowledge (Alzahrani et al. 2020). Within *Acanthus*, the species fell into two groups, one consisting of two mangrove species, and the other comprising the three non‐mangrove species. It is worthy to note that, *A. leucostachyus* showed closer relationship to other non‐mangrove species, despite being closer to mangrove species in geographic distribution. The estimation of divergence time suggested that the *Acanthus* species diverged with *Blepharis ciliaris* at approximately 16.432 Mya (95% HPD: 15.821–16.972 Mya) (Figure 8). The divergence between mangrove and non‐mangrove groups was estimated to occur at approximately 15.150 Mya (95% HPD: 14.583–15.657 Mya), and the splitting time between *A. leucostachyus* and *A. ilicifolius* was dated at approximately 0.035 Mya (95% HPD: 0.008–0.069 Mya).

## 4 Discussion

In this study, we sequenced and assembled the complete plastomes of five *Acanthus* species and performed a comprehensive comparative analysis to investigate their differences in plastome structure, CUB and evolutionary history. The phylogenetic tree reconstructed based on the plastomes revealed that, in *Acanthus*, the two mangrove species form different clades from the non‐mangrove species, although the geographic distribution of *A. leucostachyus* is closer to the two mangrove species (Figure 8). It indicates a monophyletic origin of mangrove taxa in the genus, which is consistent with our previous observation (Yang et al. 2015). The splitting time of mangrove and non‐mangrove groups was dated at approximately 15.15 Mya, which is similar to the inference based on the nuclear genome (16.8 Mya, 95% HPD: 11.6–22.1 Mya) (Yang et al. 2015). It suggests that the mangrove lineage of *Acanthus* entered intertidal habitats during the early to middle Miocene, when the earth was experiencing global warming and the sea level was in high stand (Betzler et al. 2018).

The divergence between *A. ebracteatus* and *A. ilicifolius* was supposed to occur very recently (~35 k years ago, Figure 8), which agree with their high similarities in morphology. However, the estimation is much smaller than those from nuclear DNA (He et al. 2022; Yang et al. 2015). This discrepancy might be plausibly due to cytoplasmic introgression during the hybridization of the two mangrove species (Roelofs and Bachmann 1997). *A. ebracteatus* has a nearly identical distribution with *A. ilicifolius*, suggesting a putative sympatric speciation event (Yang et al. 2015). Under this scenario, the morphological divergence between the two species might be merely controlled by a few key “speciation genes”, rather than large‐scale sequence alterations across the genome (Wu 2001). However, these highly differentiated variants might be buffered by the low‐differentiated genome background, which leads to an underestimation of divergence time between species. And the causal loci associated with the divergence of *Acanthus* mangrove species are still unknown, and more efforts are required in the future.

We then took a deep look into the structure variations among plastomes of the five *Acanthus* species. The plastome sizes of *A. ebracteatus* and *A. ilicifolius* are almost identical (150,723 and 150,791 bp). Species of the same genus or family generally have similar sizes in their plastomes. But different mangrove species have different plastome sizes, ranging from ~145 to 168 kb (Nizam et al. 2022). For instance, the plastomes of two *Acanthus* mangrove species are smaller than those from *Sonneratia* genus, *Sonneratia alba* and *S. apetala* (Ruang‐Areerate et al. 2022). It is because mangroves are a group of woody plants from 28 genera of 20 families (Duke et al. 1998), and different plastomes have different evolutionary histories, which may cause expansions/contractions of IR regions or polyphyly and finally affect the length of chloroplast DNA (Turudić et al. 2023). When comparing the sequences across plastomes, we identified regions with high sequence divergence among the five *Acanthus* species. For instance, genes *ycf1* and *psbK* were identified as the most divergent coding regions in the five *Acanthus* species (Figure 4 and Table 3), as well as in other mangrove species, *Lumnitzera littorea*, *Lu. racemosa* and *Laguncularia racemose* (Zhang et al. 2020). In addition to these two genes, *ycf2*, *rps19*, *psbK* and *ndhF* have also been reported as highly divergent genes in mangrove plastomes (Alzahrani et al. 2020; Ruang‐Areerate et al. 2021; Tan et al. 2023; Xu et al. 2021, 2022; Zhang et al. 2020; Zhang et al. 2019). For *Acanthus* species, the non‐coding regions appeared to be more variable than the coding regions (Figure 4 and Table 3), which is consistent with the observations in the mangrove plants of *Scyphiphora*, *Avicennia*, *Ceriops*, *Kandelia*, *Rhizophora*, *Bruguiera*, *Lumnitzera* and *Laguncularia* (Ruang‐Areerate et al. 2021; Tan et al. 2023; Xu et al. 2021, 2022; Zhang et al. 2020; Zhang et al. 2019). Similar to most angiosperms, the majority of these variable regions are located in the SC region, especially the LSC region (Alzahrani et al. 2020).

Compared to terrestrial non‐mangrove relatives, the two *Acanthus* mangrove species displayed special features in their plastomes, for example, specific codon usage preference and aversion (Figure 7; Tables 4 and 5). Previous studies proposed that the number of optimal codons might be correlated with a different selection mode and generally, a positive selection will result in an increased number of preferred codons, while negative selection might cause a decrease (Miller et al. 2019). Furthermore, between mangrove and non‐mangrove species, some genes, such as *pafII*, *psaJ*, *psbK*, *psbN*, *ndhH*, *rps16* and *rpl22*, were identified with signals of positive selection (Ka/Ks > 1; Figure 6). For instance, *rps16* encodes the small subunit of the ribosome and is essential for the translation accuracy and efficiency of plastid proteins, as well as the growth and survival of plants (Fleischmann et al. 2011; Keller et al. 2017). A previous study has revealed that *A. ilicifolius*, showed a better tolerance to flooding stress than its non‐mangrove relative *A. mollis*, which suffered substantial damage to xylem, phloem and periderm tissues when exposed to waterlogging (Liu and Zheng 2021). Thus, the specific amino acid composition may assist mangrove plants to cope with the environmental challenges (He et al. 2020). These findings broaden our understanding of *A. ebracteatus*’ adaptation to global climate change and assist its afforestation and restoration in the coastal wetlands of South China.

Despite the high convergence in genome sequences, we identified some regions with high sequence variations between the two close‐related mangrove species. Compared to *A. ilicifolius*, a total of 12 SNPs, eight insertions, and two deletions were detected in the plastome of *A. ebracteatus*. These sites can serve as candidate targets for designing molecular markers to distinguish the two species. PCR validation on the intergenic spacer region *a* *tpB_rbcL* in several individuals collected from wild populations revealed a high homology in its sequence within each species, while high divergence between the two groups (Figure 5), demonstrating the feasibility and effectiveness of using one molecular marker to distinguish *A. ebracteatus* from *A. ilicifolius* in the field.

## 5 Conclusion

Our current study assembled and characterized the complete plastomes of five *Acanthus* species. We showed a higher similarity in plastome sequences and structures between two mangrove species than their terrestrial non‐mangrove relatives, mirroring a monophyletic origin of mangrove plants in this genus and a very recent divergence between the two mangrove species. Seven genes related to photosystem I, photosystem II and ribosome were detected to be under positive selection, which may contribute to the different environmental adaptations between *Acanthus* mangrove and non‐mangrove plants. We further identified some variants and indels in plastomes, which can serve as informative markers for the two close‐related mangrove species. These new findings do not only offer reference data for *Acanthus* species, but also provide valuable genetic clues for the correct recognition and protection of the endangered mangrove species *A. ebracteatus*.

## Author Contributions

**Zanshan Fang:** formal analysis (equal), funding acquisition (equal), visualization (equal), writing – original draft (equal), writing – review and editing (equal). **Danfeng Li:** data curation (equal), formal analysis (equal), resources (equal), writing – review and editing (equal). **Haien Murong:** formal analysis (equal). **Meng He:** formal analysis (equal). **Yuqi Liu:** formal analysis (equal). **Jiaxuan Liu:** formal analysis (equal), visualization (equal). **Jiaxiao Wu:** formal analysis (equal). **Yingqi Li:** formal analysis (equal). **Yongyu Li:** formal analysis (equal). **Xiang Jin:** supervision (equal), writing – review and editing (equal). **Yuchen Yang:** conceptualization (equal), funding acquisition (equal), project administration (equal), resources (equal), supervision (equal), writing – original draft (equal), writing – review and editing (equal). **Ying Zhang:** conceptualization (equal), funding acquisition (equal), project administration (equal), resources (equal), supervision (lead), writing – original draft (equal), writing – review and editing (lead).

## Conflicts of Interest

The authors declare no conflicts of interest.

## Supporting information


**Data S1.** Supporting Information.

## Data Availability

The chloroplast genome sequences of *A. ebracteatus*, *A. ilicifolius*, *A. montanus*, *A. leucostachyus* and *A. mollis* were submitted to the China National GeneBank DataBase (CNGBdb), with the accession numbers of CNS0905821, CNS0905822, CNS0905825, CNS0905823 and CNS0905824. The sequencing data of PCR validation were also deposited to CNGBdb under the same project (accession number: CNP0004863).
