# Supplementary material for: Comparative Plastome Analysis Between Endangered Mangrove Species Acanthus ebracteatus and Acanthus Relatives Provides Insights into Its Origin and Adaptive Evolution
Source: Ecol Evol. 2024 Nov 20;14(11):e70566. doi: 10.1002/ece3.70566 (PMC11578654; doi:10.1002/ece3.70566)
Supplement: Supplementary file 1 — Data S1. Supporting Information. [file ECE3-14-e70566-s001.docx]

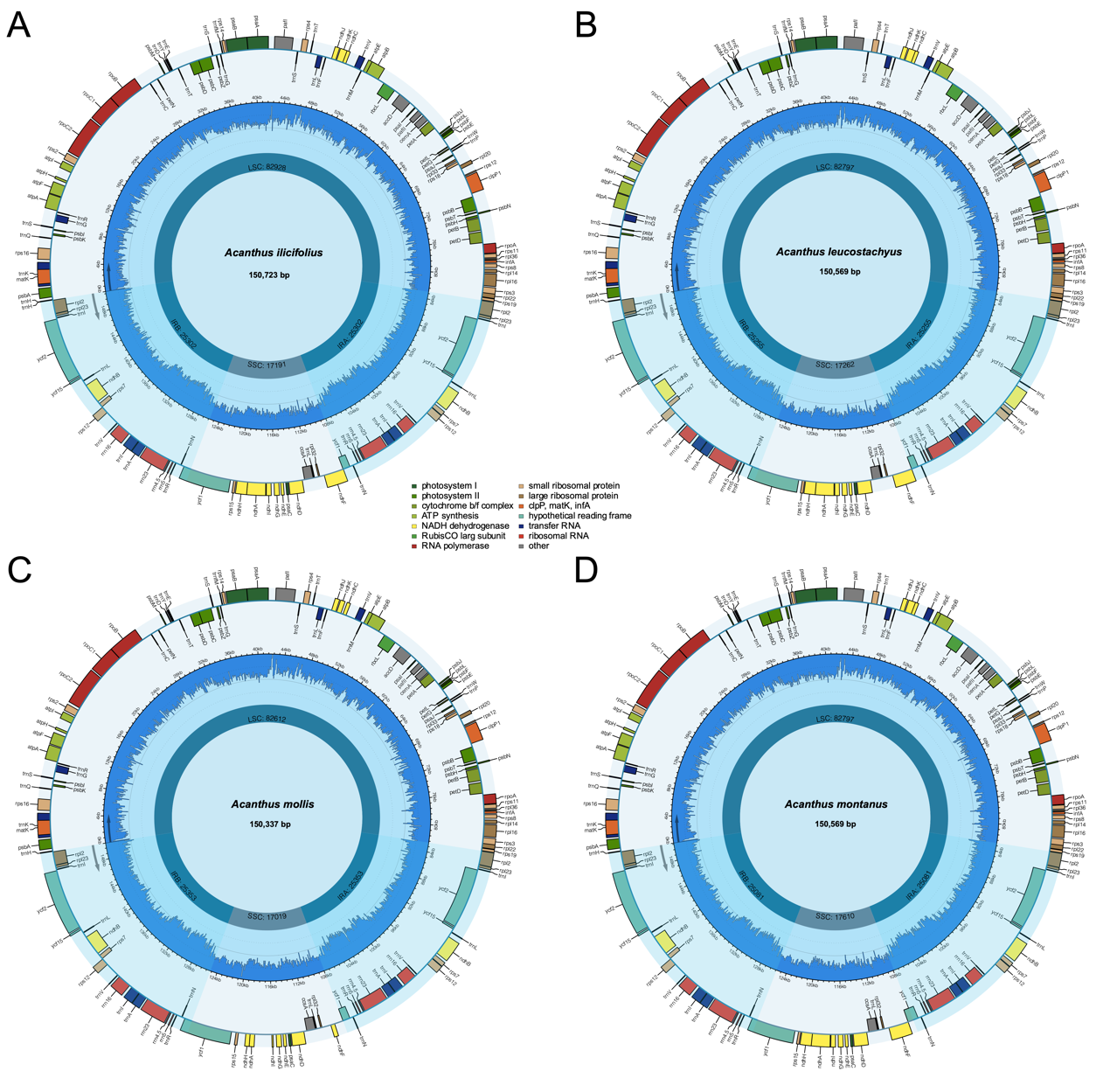


Figure S1. Chloroplast genome map of four *Acanthus* species. (A-D) Chloroplast genome map of *A. ilicifolius* (A), *A. leucostachyus* (B), *A. mollis* (C) and *A. montanus* (D). Genes transcribed clockwise and counterclockwise are listed inside and outside of the circle, respectively, where the direction is presented by arrows. The GC content of the genome is depicted as the proportion of the shaded parts of each section. Genes are color-coded by their functional classification, as listed in (A).


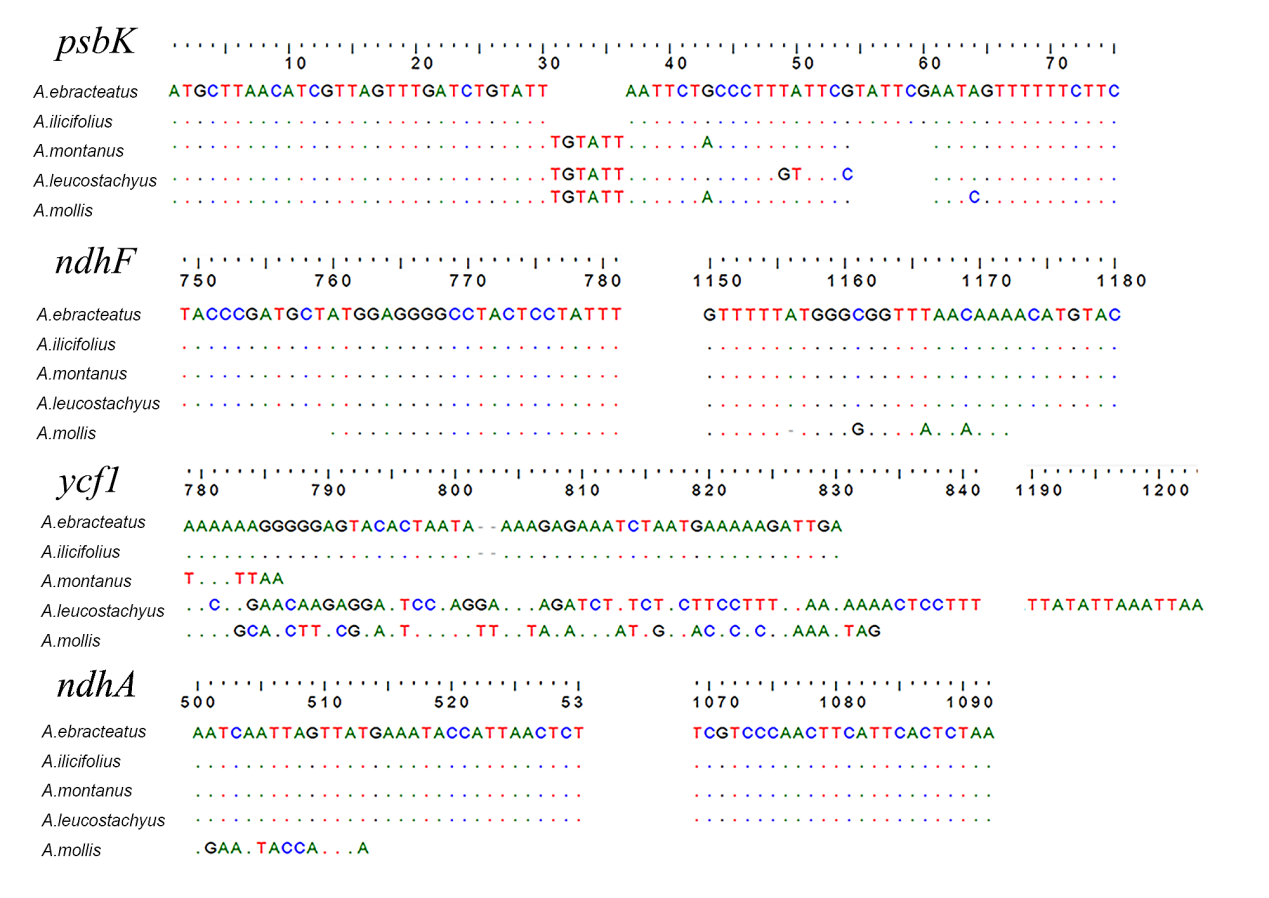


Figure S2. Substantial differences in nucleotide sequences of genes, *PsbK,* *ndhF*, *ycf1* and *ndhA*, among five *Acanthus* species.


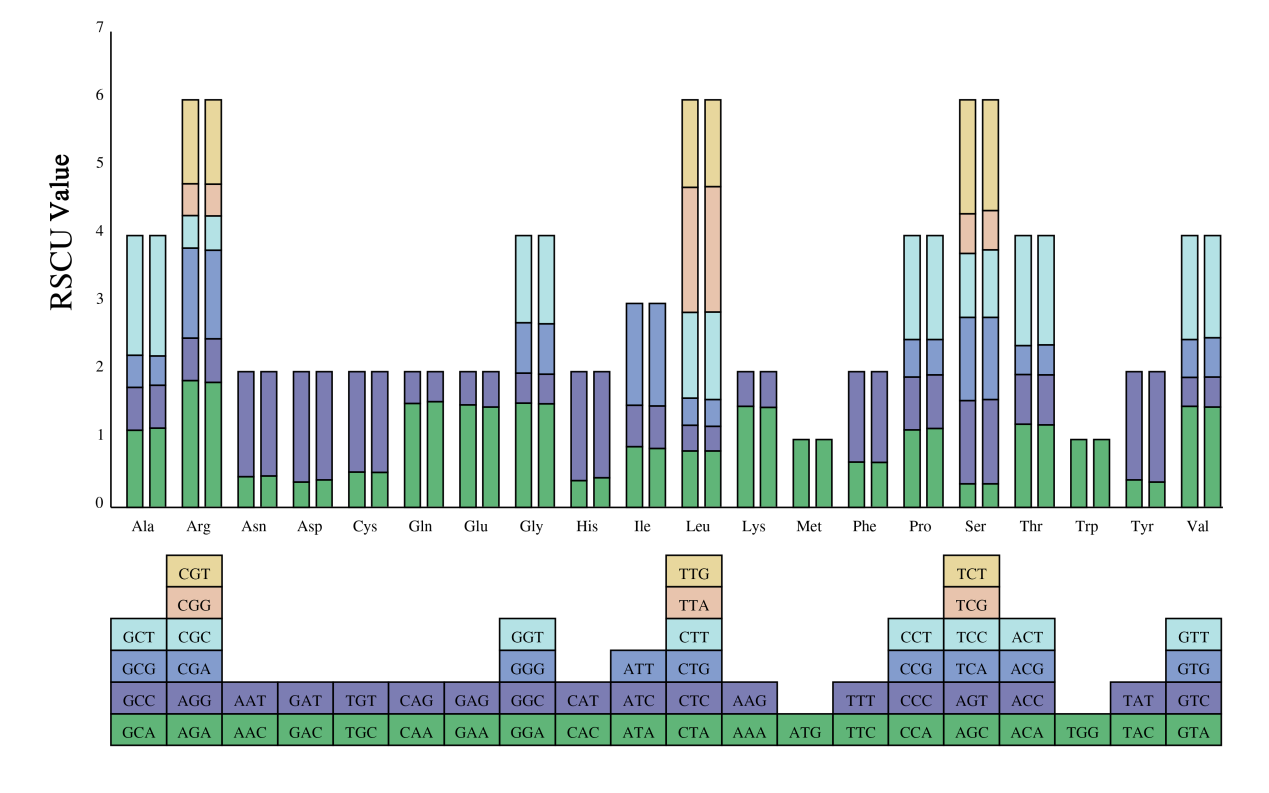


Figure S3. Codon content and RSCU value of the 20 amino acid and stop codons in all protein-coding genes of two mangrove species, *A. ilicifolius* (left column) and *A. ebracteoctus* (right column)*.* The color of the histogram corresponds to the color of codons.


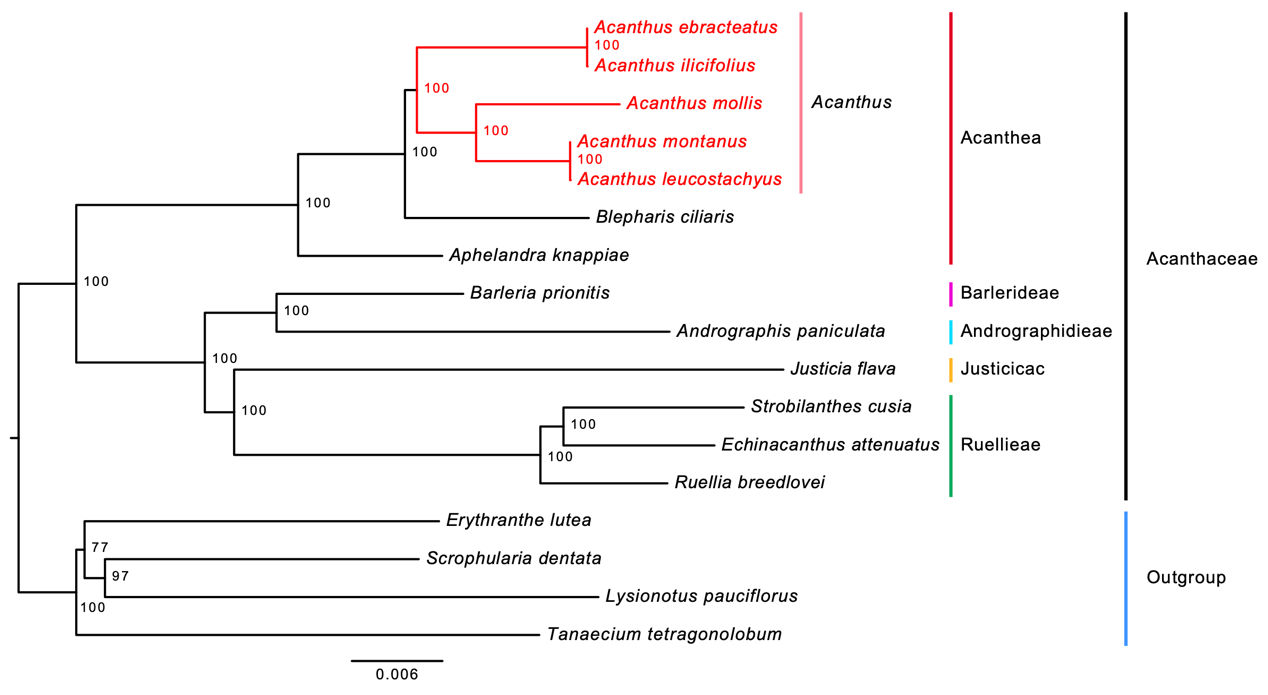


Figure S4. Maximum likelihood tree of Acantheae species constructed using the orthologous chloroplast coding sequences. Species of *Acanthus* are highlighted in red. Numbers at the nodes indicate maximum likelihood bootstrap values.

Table S1. Sequences, annealing temperature (Tm), and concentration of each pair of primers used in this study.

| Gene | Species | Forward primer | Reverse primer | Tm | Concentration |
| --- | --- | --- | --- | --- | --- |
| *psbK* | *A.ebracteatus* | ATGCTTAACATCGTTAGTTTGATCT | TCATCGAAAACTTACAGCGGC | 57℃ | 0.2μM |
|  | *A. ilicifolius* | ATGCTTAACATCGTTAGTTTGATCT | TCATCGAAAACTTACAGCGGC | 57℃ | 0.2μM |
|  | *A.leucostachyus* | ATGCTTAACATCGTTAGTTTGATCT | TCATCGAAAACTTACAGCGGC | 57℃ | 0.2μM |
|  | *A.montanus* | ATGCTTAACATCGTTAGTTTGATCT | TCATCGAAAACTTACAGCGGC | 57℃ | 0.2μM |
|  | *A. mollis* | ATGCTTAACATCGTTAGTTTGATCT | TCATCGAAAACTTACAGCGGC | 57℃ | 0.2μM |
| *ndhF* | *A.ebracteatus* | ATGGAACAGACATATCAATATGTAT | KTAWATTAATATTAATAAAAAAATGAATAC | 60℃ | 0.2μM |
|  | *A. ilicifolius* | ATGGAACAGACATATCAATATGTAT | KTAWATTAATATTAATAAAAAAATGAATAC | 60℃ | 0.2μM |
|  | *A.leucostachyus* | ATGGAACAGACATATCAATATGTAT | KTAWATTAATATTAATAAAAAAATGAATAC | 60℃ | 0.2μM |
|  | *A.montanus* | ATGGAACAGACATATCAATATGTAT | KTAWATTAATATTAATAAAAAAATGAATAC | 60℃ | 0.2μM |
|  | *A. mollis* | ATGGAACAGACATATCAATATGTAT | KTAWATTAATATTAATAAAAAAATGAATAC | 60℃ | 0.2μM |
| *ndhA* | *A.ebracteatus* | ATGATAATTGATACAACCGAAATAC | TTAGAGTGAATGAAGTTGGGAC | 57℃ | 0.2μM |
|  | *A. ilicifolius* | ATGATAATTGATACAACCGAAATAC | TTAGAGTGAATGAAGTTGGGAC | 57℃ | 0.2μM |
|  | *A.leucostachyus* | ATGATAATTGATACAACCGAAATAC | TTAGAGTGAATGAAGTTGGGAC | 57℃ | 0.2μM |
|  | *A.montanus* | ATGATAATTGATACAACCGAAATAC | TTAGAGTGAATGAAGTTGGGAC | 57℃ | 0.2μM |
|  | *A. mollis* | ATGATAATTGATACAACCAAAATAC | TTAATGGTATTTCTGAGCAGC | 57℃ | 0.2μM |
| *ycf1* | *A.ebracteatus* | ATGATTTTTCAATCTTTTCTACT | TCAATCTTTTTCATTAGATTTCTCT | 54℃ | 0.2μM |
|  | *A. ilicifolius* | ATGATTTTTCAATCTTTTCTACT | TCAATCTTTTTCATTAGATTTCTCT | 54℃ | 0.2μM |
|  | *A.leucostachyus* | ATGATTTTTCAATCTTTTCTACT | TTAATTTAATATAATATTTAATCTGTTTTC | 58℃ | 0.2μM |
|  | *A.montanus* | ATGATTTTTCAATCTTTTCTACT | TTAATTTAATATAATATTTAATCTGTTTTC | 58℃ | 0.2μM |
|  | *A. mollis* | ATGATTTTTCAATCTTTTCTACT | CTATTTTTCGTGTGTATCA | 54℃ | 0.2μM |
| *atpB_rbcL* | *A.ebracteatus*  *A. ilicifolius* | AGCACTCGATTTTGTTGTGCC | ATAGCGCAACCCCATTTCCC | 55 ℃ | 0.2μM |

Table S2. Accession numbers of published chloroplast genomes used in this study

| Species | Accession number | Database |
| --- | --- | --- |
| *Acanthus ebracteatus* | CNS0905821 | CNGBdb |
| *Acanthus ilicifolius* | CNS0905822 |  |
| *Acanthus leucostachyus* | CNS0905823 |  |
| *Acanthus montanus* | CNS0905824 |  |
| *Acanthus mollis* | CNS0905825 |  |
| *Blepharis ciliaris* | NC_046601.1 | NCBI |
| *Aphelandra knappiae* | NC_041424.1 |  |
| *Andrograhis paniculata* | NC_022451.2 |  |
| *Barleria prionitis* | MK548575.1 |  |
| *Echinacanthus attenuatus* | NC_039762.1 |  |
| *Strobilanthes cusia* | NC_037485.1 |  |
| *Ruellia breedlovei* | KP300014.1 |  |
| *Justicia flava* | NC_044862.1 |  |
| *Erythranthe lutea* | NC_030212.1 |  |
| *Lysionotus pauciflorus* | NC_034660.1 |  |
| *Scrophularia dentata* | NC_036942.1 |  |
| *Tanaecium tetragonolobum* | NC_027955.1 |  |

Table S3. the SNPs and indels in the chloroplast (cp) genome of *Acanthus ebracteatus* versus *A. ilicifolius*

|  | No. | Position in *A. ebracteatus* cp genome | Allele of *A. ebracteatus* | Position in *A. ilicifolius* cp genome | Allele of *A. ilicifolius* | Gene_id |
| --- | --- | --- | --- | --- | --- | --- |
| SNPs | 1 | 6546 | T | 6544 | G | *rps16-trnQ-UUG* |
|  | 2 | 24066 | A | 24064 | C | *rpoB* |
|  | 3 | 46698 | A | 46658 | T | *trnT-UGU-trnL-UAA* |
|  | 4 | 46700 | A | 46660 | T | *trnT-UGU-trnL-UAA* |
|  | 5 | 46708 | A | 46665 | T | *trnT-UGU-trnL-UAA* |
|  | 6 | 46715 | T | 46672 | A | *trnT-UGU-trnL-UAA* |
|  | 7 | 46717 | T | 46674 | A | *trnT-UGU-trnL-UAA* |
|  | 8 | 54385 | A | 54341 | C | *atpB-rbcL* |
|  | 9 | 72975 | T | 72913 | G | *psbB-psbT* |
|  | 10 | 108471 | A | 108406 | C | *ndhF* |
|  | 11 | 116214 | A | 116149 | G | *ndhG-ndhI* |
|  | 12 | 125377 | A | 125312 | G | *ycf1* |
|  | 1 | 4446 | TTC | 4446 | T | *matK-rps16* |
| Indels | 2 | 44472 | ATTAATGATAGTAAAGAAGAAA | 44470 | A | *paf1-trns-GGA* |
|  | 3 | 46671 | TATAATAATATATATATA | 46648 | T | *trnT-UGU-trnL-UAA* |
|  | 4 | 46703 | TATT | 46663 | T | *trnT-UGU-trnL-UAA* |
|  | 5 | 46719 | TA | 46676 | T | *trnT-UGU-trnL-UAA* |
|  | 6 | 54649 | TATTCTTATTATAGAAAAT | 54605 | T | *atpB-rbcL* |
|  | 7 | 95260 | G | 95198 | GGTATAT | *ndhB* |
|  | 8 | 102793 | ATACTTATAA | 102737 | A | *trnA-UGC-rrn23* |
|  | 9 | 130983 | TAAGTATTAT | 130918 | T | *rrn23--trnA-UGC* |
|  | 10 | 138521 | G | 138447 | GATATAC | *ndhB* |

Table S4. Codon usage and codon-anticodon recognition patterns in the chloroplast genomes of five *Acanthus* species

| Codon | Numbers/RSCU | | | | |
| --- | --- | --- | --- | --- | --- |
|  | *Acanthus ilicifolius* | *Acanthus ebracteatus* | *Acanthus montanus* | *Acanthus leucostachyus* | *Acanthus mollis* |
| AAA(K) | 753/1.4736 | 753/1.4736 | 783/1.4928 | 794/1.4882 | 771/1.4884 |
| AAC(N) | 219/0.465 | 218/0.4628 | 216/0.4556 | 218/0.4536 | 215/0.4654 |
| AAG(K) | 269/0.5264 | 269/0.5264 | 266/0.5072 | 273/0.5118 | 265/0.5116 |
| AAU(N) | 723/1.535 | 724/1.5372 | 732/1.5444 | 743/1.5464 | 709/1.5346 |
| ACA(T) | 308/1.2172 | 308/1.2172 | 312/1.2296 | 313/1.2252 | 283/1.188 |
| ACC(T) | 186/0.7352 | 186/0.7352 | 185/0.7292 | 187/0.732 | 166/0.6968 |
| ACG(T) | 111/0.4388 | 111/0.4388 | 108/0.4256 | 108/0.4228 | 108/0.4532 |
| ACU(T) | 407/1.6088 | 407/1.6088 | 410/1.6156 | 414/1.6204 | 396/1.662 |
| AGA(R) | 360/1.8384 | 360/1.8384 | 372/1.8522 | 378/1.8666 | 361/1.8816 |
| AGC(S) | 86/0.3486 | 86/0.3486 | 87/0.3462 | 88/0.3462 | 86/0.3606 |
| AGG(R) | 126/0.6432 | 126/0.6432 | 128/0.6372 | 127/0.627 | 125/0.6516 |
| AGU(S) | 306/1.2396 | 306/1.2396 | 311/1.2366 | 312/1.2282 | 293/1.2294 |
| AUA(I) | 484/0.8706 | 483/0.8691 | 507/0.8904 | 512/0.8931 | 481/0.8814 |
| AUC(I) | 347/0.624 | 347/0.6246 | 349/0.6129 | 350/0.6105 | 343/0.6285 |
| AUG(M) | 449/1 | 449/1 | 457/1 | 458/1 | 421/1 |
| AUU(I) | 837/1.5054 | 837/1.5063 | 852/1.4964 | 858/1.4964 | 813/1.4898 |
| CAA(Q) | 563/1.5596 | 563/1.5596 | 576/1.5298 | 578/1.5292 | 552/1.529 |
| CAC(H) | 102/0.4368 | 102/0.4368 | 95/0.3958 | 95/0.395 | 95/0.4068 |
| CAG(Q) | 159/0.4404 | 159/0.4404 | 177/0.4702 | 178/0.4708 | 170/0.471 |
| CAU(H) | 365/1.5632 | 365/1.5632 | 385/1.6042 | 386/1.605 | 372/1.5932 |
| CCA(P) | 234/1.1628 | 234/1.164 | 239/1.1448 | 240/1.14 | 218/1.1192 |
| CCC(P) | 158/0.7852 | 158/0.786 | 163/0.7808 | 164/0.7792 | 157/0.806 |
| CCG(P) | 105/0.5216 | 105/0.5224 | 114/0.546 | 116/0.5512 | 107/0.5496 |
| CCU(P) | 308/1.5304 | 307/1.5272 | 319/1.528 | 322/1.5296 | 297/1.5252 |
| CGA(R) | 255/1.302 | 255/1.302 | 266/1.3242 | 268/1.3236 | 252/1.3134 |
| CGC(R) | 99/0.5058 | 99/0.5058 | 95/0.4728 | 97/0.4788 | 89/0.4638 |
| CGG(R) | 92/0.4698 | 92/0.4698 | 93/0.4632 | 94/0.4644 | 86/0.4482 |
| CGU(R) | 243/1.2408 | 243/1.2408 | 251/1.2498 | 251/1.2396 | 238/1.2408 |
| CUA(L) | 284/0.8334 | 284/0.8334 | 293/0.8364 | 294/0.8334 | 262/0.8034 |
| CUC(L) | 124/0.3636 | 124/0.3636 | 131/0.3738 | 132/0.3744 | 120/0.3678 |
| CUG(L) | 134/0.393 | 134/0.393 | 141/0.4026 | 142/0.4026 | 143/0.4386 |
| CUU(L) | 438/1.2852 | 438/1.2852 | 438/1.2504 | 445/1.2618 | 415/1.2732 |
| GAA(E) | 748/1.4782 | 748/1.4782 | 787/1.5076 | 797/1.511 | 745/1.493 |
| GAC(D) | 161/0.4036 | 161/0.404 | 153/0.3732 | 155/0.3766 | 140/0.3664 |
| GAG(E) | 264/0.5218 | 264/0.5218 | 257/0.4924 | 258/0.489 | 253/0.507 |
| GAU(D) | 637/1.5964 | 636/1.596 | 667/1.6268 | 668/1.6234 | 624/1.6336 |
| GCA(A) | 316/1.1716 | 316/1.1704 | 313/1.1412 | 313/1.1392 | 301/1.1444 |
| GCC(A) | 169/0.6264 | 169/0.626 | 171/0.6236 | 172/0.626 | 173/0.6576 |
| GCG(A) | 117/0.4336 | 117/0.4332 | 131/0.4776 | 131/0.4768 | 119/0.4524 |
| GCU(A) | 477/1.7684 | 478/1.7704 | 482/1.7576 | 483/1.758 | 459/1.7452 |
| GGA(G) | 508/1.528 | 508/1.528 | 522/1.5352 | 522/1.534 | 485/1.5144 |
| GGC(G) | 145/0.436 | 145/0.436 | 150/0.4412 | 151/0.4436 | 150/0.4684 |
| GGG(G) | 246/0.74 | 245/0.7368 | 251/0.7384 | 252/0.7408 | 242/0.7556 |
| GGU(G) | 431/1.2964 | 432/1.2992 | 437/1.2852 | 436/1.2816 | 404/1.2616 |
| GUA(V) | 394/1.4784 | 394/1.48 | 409/1.486 | 411/1.4892 | 384/1.4768 |
| GUC(V) | 118/0.4428 | 117/0.4396 | 118/0.4288 | 118/0.4276 | 107/0.4116 |
| GUG(V) | 154/0.578 | 154/0.5784 | 152/0.5524 | 153/0.5544 | 161/0.6192 |
| GUU(V) | 400/1.5008 | 400/1.5024 | 422/1.5332 | 422/1.5288 | 388/1.4924 |
| UAA(*) | 28/1.7142 | 28/1.7142 | 28/1.7142 | 28/1.7142 | 28/1.7142 |
| UAC(Y) | 135/0.375 | 135/0.375 | 146/0.3978 | 150/0.406 | 131/0.3814 |
| UAG(*) | 9/0.5511 | 9/0.5511 | 10/0.6123 | 10/0.6123 | 9/0.5511 |
| UAU(Y) | 585/1.625 | 585/1.625 | 588/1.6022 | 589/1.594 | 556/1.6186 |
| UCA(S) | 299/1.2114 | 299/1.2114 | 309/1.2288 | 311/1.2246 | 278/1.1664 |
| UCC(S) | 245/0.9924 | 245/0.9924 | 236/0.9384 | 239/0.9408 | 239/1.0026 |
| UCG(S) | 142/0.5754 | 142/0.5754 | 146/0.5808 | 147/0.579 | 151/0.6336 |
| UCU(S) | 403/1.6326 | 403/1.6326 | 420/1.6698 | 427/1.6812 | 383/1.6068 |
| UGA(*) | 12/0.7347 | 12/0.7347 | 11/0.6735 | 11/0.6735 | 12/0.7347 |
| UGC(C) | 54/0.5168 | 54/0.5168 | 57/0.523 | 57/0.5206 | 55/0.5418 |
| UGG(W) | 347/1 | 347/1 | 358/1 | 360/1 | 328/1 |
| UGU(C) | 155/1.4832 | 155/1.4832 | 161/1.477 | 162/1.4794 | 148/1.4582 |
| UUA(L) | 629/1.8456 | 629/1.8456 | 646/1.8438 | 648/1.8372 | 596/1.8282 |
| UUC(F) | 363/0.6606 | 364/0.6618 | 368/0.6716 | 371/0.6708 | 332/0.6594 |
| UUG(L) | 436/1.2792 | 436/1.2792 | 453/1.293 | 455/1.29 | 420/1.2882 |
| UUU(F) | 736/1.3394 | 736/1.3382 | 728/1.3284 | 735/1.3292 | 675/1.3406 |

Table S5. Codon aversion pattern of the shared CDSs in five *Acanthus* chloroplast genomes

| Gene | Species | Codons |
| --- | --- | --- |
| *accD* | Ae | CUC、CCG、UAG、UGA、CGA |
|  | Ai | UGA、UAA、CUC |
|  | Al | UGA、UAA、CUC |
|  | Aml | CUC、UAG、UGA |
|  | Amn | UGA、UAA、CUC |
| *atpA* | Ae | UAG、UGC、UGA、UGG |
|  | Ai | UGC、UGA、UGG、AGC、UAG、AAC |
|  | Al | UGC、UGA、UGG、AGC、UAG、AAC |
|  | Aml | CCG、UAG、AAC、UGC、UGA、UGG、AGC |
|  | Amn | UGC、UGA、UGG、AGC、UAG、AAC |
| *atpB* | Ae | CUC、UAA、UAG、UGC、UGG |
|  | Ai | UGC、UGG、UAA、UAG、CAC |
|  | Al | UGC、UGG、UAA、UAG、CAC |
|  | Aml | UAA、UAG、CAC、UGC、UGG |
|  | Amn | UGC、UGG、UAA、UAG、CAC |
| *atpE* | Ae | UUC、UCC、UCG、CCC、CCA、CCG、UAU、UAC、UAG、CAC、UGC、UGA、CGU、CGG、AGC |
|  | Ai | UUC、UGC、UGA、CGU、CGG、AGC、UAU、UAC、UAG、CAC、UCC、UCG、CCC、CCA、CCG |
|  | Al | UUC、UGC、UGA、CGU、CGG、AGC、UAU、UAC、UAG、CAC、UCC、UCG、CCC、CCA、CCG |
|  | Aml | UUC、UCC、UCG、CCC、CCA、UAU、UAC、UAG、CAC、UGC、UGA、CGU、CGG、AGC |
|  | Amn | UGC、UGA、CGU、CGG、AGC、UAU、UAC、UAG、CAC、UCC、UCG、CCC、CCA、CCG、UUC |
| *atpF* | Ae | CUU、CUC、UCC、CCU、CCC、CCG、UAA、UGC、UGA、AGC |
|  | Ai | UGC、UGA、AGC、UAA、GAC、UCC、CCU、CCC、CCG、CUU、CUC、GUC |
|  | Al | UGC、UGA、AGC、UAA、GAC、UCC、CCU、CCC、CCG、CUU、CUC、GUC |
|  | Aml | CUU、CUC、UCC、CCU、CCC、CCG、UAA、GAC、UGC、UGA、AGC |
|  | Amn | UGC、UGA、AGC、UAA、GAC、UCC、CCU、CCC、CCG、CUU、CUC、GUC |
| *atpI* | Ae | CUG、UCG、UAA、UAG、GAC、UGC、CGU、CGC、AGC、AGA |
|  | Ai | UGC、CGU、CGC、AGC、AGA、AGG、UAA、UAG、CAC、GAC、UCG、CUG |
|  | Al | UGC、CGU、CGC、AGC、AGA、AGG、UAA、UAG、CAC、GAC、UCG、CUG |
|  | Aml | CUG、UCG、UAA、UAG、CAC、GAC、UGC、CGU、CGC、AGC、AGA、AGG |
|  | Amn | UGC、CGU、CGC、AGC、AGA、AGG、UAA、UAG、CAC、GAC、UCG、CUG |
| *ccsA* | Ae | CCC、CCG、UAA、UAG、CGC、AGC |
|  | Ai | CCC、CCG、CUG、CGC、AGC、AGG、UAA、UAG、CAC |
|  | Al | CCC、CCG、CUG、CGC、AGC、AGG、UAA、UAG、CAC |
|  | Aml | CUG、CCC、CCG、UAA、UAG、CGC、AGC |
|  | Amn | CGC、AGC、AGG、UAA、UAG、CAC、CCC、CCG、CUG |
| *cemA* | Ae | CUG、GCC、UAA、UAG、UGC、CGC、CGA、CGG、AGA、GGC、GGG |
|  | Ai | UGC、CGC、CGA、CGG、AGA、AGG、GGC、GGG、UAA、UAG、GCC、GCG |
|  | Al | UGC、CGC、CGA、CGG、AGA、AGG、GGC、GGG、UAA、UAG、GCC、GCG |
|  | Aml | GCC、GCG、UAA、UAG、UGC、CGC、CGA、CGG、AGG、GGC、GGG |
|  | Amn | UGC、CGC、CGA、CGG、AGA、AGG、GGC、GGG、UAA、UAG、GCC、GCG |
| *clpP1* | Ae | GUC、UCC、UCG、CCG、UAA、UAG、CAG、AAG、UGU、CGG |
|  | Ai | UGU、AAG、CGG、UAA、UAG、UCC、UCG、CCC、GUC |
|  | Al | UGU、AAG、CGG、UAA、UAG、UCC、UCG、CCC、GUC |
|  | Aml | GUC、UCC、UCG、UAA、UAG、AAG、UGU、CGG |
|  | Amn | UGU、CGG、UAA、UAG、AAG、UCC、UCG、CCC、GUC |
| *matK* | Ae | UAA、UAG |
|  | Ai | UAA、UAG |
|  | Al | UAA、UAG |
|  | Aml | UAC、UAA、UAG |
|  | Amn | UAA、UAG |
| *ndhA* | Ae | CCG、ACG、UAG、CAC、CAG、UGC、UGA、CGA、GGC |
|  | Ai | UGC、UGA、UAG、CAC、CCG、GGC |
|  | Al | UGC、UGA、UAG、CAC、CCG、GGC |
|  | Aml | CCG、ACG、GCG、UAG、CAC、GAC、GAG、UGU、UGC、UGA、CGA、GGC |
|  | Amn | UGC、UGA、GGC、UAG、CAC、CCG |
| *ndhB* | Ae | UAA、UGA、CGC、AGG |
|  | Ai | UGA、CGC、AGG、UAA |
|  | Al | UGA、CGC、AGG、UAA |
|  | Aml | UAA、UGA、CGC、AGG |
|  | Amn | UGA、CGC、AGG、UAA |
| *ndhC* | Ae | CUC、CUG、GUC、GUG、CCC、ACC、ACG、GCG、UAA、CAU、CAC、CAG、AAU、GAC、UGU、UGC、UGA、CGC、CGG、AGC、AGA、AGG、GGC |
|  | Ai | UGU、UGC、UGA、CGC、CGG、AGC、UAA、CAU、CAC、CAG、AAC、GAC、CCC、ACC、ACA、GCG、CUC、CUG、GUC、GUG |
|  | Al | UGU、UGC、UGA、CGC、CGG、AGC、UAA、CAU、CAC、CAG、AAC、GAC、CCC、ACC、ACA、GCG、CUC、CUG、GUC、GUG |
|  | Aml | CUC、CUG、GUC、GUG、CCC、ACC、ACG、GCG、UAA、CAU、CAC、CAG、AAU、GAC、UGU、UGC、UGA、CGC、CGG、AGC、AGG |
|  | Amn | UGU、UGC、UGA、CGC、CGG、AGC、UAA、CAU、CAC、CAG、AAC、GAC、CCC、ACC、ACA、GCG、CUC、CUG、GUC、GUG |
| *ndhD* | Ae | UAA、CAC、UGC、UGA、AGC、AGG |
|  | Ai | UGC、UGA、AGC、AGG、UAA、CAC |
|  | Al | UGC、UGA、AGC、AGG、UAA、CAC |
|  | Aml | UAA、CAG、UGC、UGA、AGG |
|  | Amn | UGC、UGA、AGC、AGG、UAA、CAC |
| *ndhE* | Ae | CUA、CUG、GUG、UCG、CCU、CCC、CCA、ACU、ACC、ACG、GCG、UAC、UAG、CAC、CAG、GAG、UGC、UGA、UGG、CGG、AGC、GGC、GGG |
|  | Ai | UGC、UGA、UGG、CGC、CGG、AGC、GGC、GGG、UAC、UAG、CAC、CAG、AAG、GAG、UCG、CCU、CCC、CCA、AC、GCG、CUA、GUG |
|  | Al | UGC、UGA、UGG、CGC、CGG、AGC、GGC、GGG、UAC、UAG、CAC、CAG、AAG、GAG、UCG、CCU、CCC、CCA、AC、GCG、CUA、GUG |
|  | Aml | CUA、GUG、UCG、CCU、CCC、CCA、ACU、ACC、ACG、GCG、UAC、UAG、CAC、CAG、AAG、GAG、UGC、UGA、UGG、CGC、CGG、AGC、GGC、GGG |
|  | Amn | UGC、UGA、UGG、CGC、CGG、AGC、GGC、GGG、UAC、UAG、CAC、CAG、AAG、GAG、UCG、CCU、CCC、CCA、ACC、GCG、CUA、GUG |
| *ndhF* | Ae | UAA、UGC、UGA |
|  | Ai | UGC、UGA、CGC、UAG、CUG |
|  | Al | UGC、UGA、CGC、UAG、CUG |
|  | Aml | CUC、CUG、GUC、GUG、CCC、CCA、UAC、UAA、CAC、AAC、AAG、UGU、UGC、UGA、UGG、CGU、CGG、AGU、AGC、AGG |
|  | Amn | UGC、UGA、CGC、UAG、CUG |
| *ndhG* | Ae | UCU、CCC、GCG、UAC、UAG、CAC、AAG、GAC、GAG、UGA、CGC、CGG、AGC、AGG、GGC |
|  | Ai | UGC、UGA、CGC、CGA、CGG、AGC、AGG、GGC、UAG、CAC、AAG、GAC、GAG、UCU、CCC、CCG、ACG |
|  | Al | UGC、UGA、CGC、CGA、CGG、AGC、AGG、GGC、UAG、CAC、AAG、GAC、GAG、UCU、CCC、CCG、ACG |
|  | Aml | CUC、UCU、CCC、CCG、UAG、CAC、AAG、GAC、GAG、UGC、UGA、CGC、CGG、AGC、AGA、AGG、GGC |
|  | Amn | UGC、UGA、CGC、CGA、CGG、AGC、AGG、GGC、UAG、CAC、AAG、GAC、GAG、UCU、CCC、CCG、ACG |
| *ndhH* | Ae | UAC、UAA、UAG |
|  | Ai | UAC、UAA、UAG、GUG |
|  | Al | UAC、UAA、UAG、GUG |
|  | Aml | UUC、CUC、GUG、CCC、ACC、GCG、UAC、UAA、UAG、CAU、CAC、AAG、UGU、UGC |
|  | Amn | UAC、UAA、UAG、GUG |
| *ndhJ* | Ae | CUC、UCA、UCG、ACA、ACG、GCG、UAA、UAG、CAC、AAC、GAC、CGG |
|  | Ai | CGG、UAA、UAG、CAC、AAC、GAC、UCA、UCG、ACA、ACG、GCG、CUC |
|  | Al | CGG、UAA、UAG、CAC、AAC、GAC、UCA、UCG、ACA、ACG、GCG、CUC |
|  | Aml | UCA、ACA、ACG、GCG、UAA、UAG、CAC、AAC、GAC、CGG |
|  | Amn | CGG、UAA、UAG、CAC、AAC、GAC、UCA、UCG、ACA、ACG、GCG、CUC |
| *ndhK* | Ae | CUG、ACG、UAA、CAG、AAC、UGC、UGA |
|  | Ai | UGA、CGC、UAC、UAG、CAG、ACG、CUG |
|  | Al | UGA、CGC、UAC、UAG、CAG、ACG、CUG |
|  | Aml | CUG、UCA、ACG、UAC、UAG、CAG、AAC、GAC、UGC、UGA、UGG、CGC、GGU |
|  | Amn | UGA、CGC、UAC、UAG、CAG、ACG、CUG |
| *pafI* | Ae | CUG、GUU、GUA、UCU、CCC、UAG、CAC、AAC、GAC、UGC、UGA、AGC、AGG |
|  | Ai | UGC、UGA、AGC、AGG、GGC、UAG、CAC、AAC、UCU、CCC、CUG、GUU、GUA |
|  | Al | UGC、UGA、AGC、AGG、GGC、UAG、CAC、AAC、UCU、CCC、CUG、GUU、GUA |
|  | Aml | CUG、GUU、GUA、UCU、CCC、UAG、CAC、AAC、UGC、UGA、AGC、AGG |
|  | Amn | UGC、UGA、AGC、AGG、GGC、UAG、CAC、AAC、UCU、CCC、CUG、GUU、GUA |
| *pafII* | Ae | CUG、UCG、UAA、UAG、CAC、AAC、AGC、AGG |
|  | Ai | AGC、AGG、AAC、CAC、UAG、UAA、UCG、CCC、CUA、CUG |
|  | Al | AGC、AGG、AAC、CAC、UAG、UAA、UCG、CCC、CUA、CUG |
|  | Aml | CUG、UCG、CCC、UAA、UAG、CAC、AGC、AGG |
|  | Amn | AGC、AGG、UAA、UAG、CAC、AAC、UCG、CCC、CUA、CUG |
| *petA* | Ae | CUA、CUG、UAA、UGC、UGA、CGC |
|  | Ai | UGC、UGA、CGC、UAC、UAA、CUA、CUG |
|  | Al | UGC、UGA、CGC、UAC、UAA、CUA、CUG |
|  | Aml | CUG、UAA、UGC、UGA、CGC |
|  | Amn | UGC、UGA、CGC、UAC、UAA、CUA、CUG |
| *petB* | Ae | CUG、UAA、AAC、AAG、UGC、UGA、AGA、AGG、GGC、 |
|  | Ai | UGC、UGA、CGG、AGA、AGG、GGC、UAA、AAC、AAG、CUG |
|  | Al | UGC、UGA、CGG、AGA、AGG、GGC、UAA、AAC、AAG、CUG |
|  | Aml | UAA、AAC、AAG、UGC、UGA、CGG、AGA、AGG、GGC |
|  | Amn | UGC、UGA、CGG、AGA、AGG、GGC、UAA、AAC、AAG、CUG |
| *petD* | Ae | CUC、CUG、GUG、UCU、UCG、ACG、UAG、CAC、CAG、AAG、GAC、UGC、UGA、CGA、CGG、AGU、AGC、AGG、GGG |
|  | Ai | UGC、UGA、CGA、CGG、AGU、AGC、AGG、GGG、UAG、CAC、CAG、AAG、GAC、UCU、CCG、ACG、CUC、CUG |
|  | Al | UGC、UGA、CGA、CGG、AGU、AGC、AGG、GGG、UAG、CAC、CAG、AAG、GAC、UCU、CCG、ACG、CUC、CUG |
|  | Aml | CUC、CUG、UCU、CCG、ACG、UAC、UAG、CAC、CAG、AAG、GAC、UGC、UGA、CGA、CGG、AGU、AGC、AGG、GGG |
|  | Amn | UGC、UGA、CGA、CGG、AGU、AGC、AGG、GGG、UAG、CAC、CAG、AAG、GAC、UCU、CCG、ACG、CUC、CUG |
| *psaA* | Ae | UAG、UGC、UGA |
|  | Ai | UGC、UGA、UAG |
|  | Al | UGC、UGA、UAG |
|  | Aml | UAG、UGC、UGA |
|  | Amn | UGC、UGA、UAG |
| *psaB* | Ae | UAG、UGA、CGG |
|  | Ai | UGA、UAG |
|  | Al | UGA、UAG |
|  | Aml | UAG、UGA、CGG |
|  | Amn | UGA、UAG |
| *psbA* | Ae | CCC、CCG、ACG、GCG、UAG、AAA、AAG、UGC、UGA、CGG |
|  | Ai | UGC、UGA、UAG、CUC、CCC、CCG、CGG、AAA、AAG、ACG、GCG |
|  | Al | UGC、UGA、UAG、CUC、CCC、CCG、CGG、AAA、AAG、ACG、GCG |
|  | Aml | CUC、CCC、CCG、ACG、GCG、UAG、AAA、AAG、UGC、UGA、CGG |
|  | Amn | UGU、UGC、UGA、CGG、UAU、UAG、AAA、AAG、CCC、CCG、ACG、CUC |
| *psbB* | Ae | UAA、UAG |
|  | Ai | UAA、UAG |
|  | Al | UAA、UAG |
|  | Aml | UAA、UAG |
|  | Amn | UAA、UAG |
| *psbC* | Ae | UCA、UCG、UAA、UAG、UGC、CGC |
|  | Ai | UGC、CGC、UAA、UAG、UCA、UCG |
|  | Al | UGC、CGC、UAA、UAG、UCA、UCG |
|  | Aml | UCA、UCG、UAA、UAG、UGC、CGC |
|  | Amn | UGC、CGC、UAA、UAG、UCA、UCG |
| *psbD* | Ae | GUG、UCA、CCC、UAC、UAG、CAC、AAG、UGA、AGC、AGG |
|  | Ai | UGA、AGC、AGG、UAC、UAG、CAC、AAG、UCG、CCC、GUG |
|  | Al | UGA、AGC、AGG、UAC、UAG、CAC、AAG、UCG、CCC、GUG |
|  | Aml | GUG、UCG、CCC、UAC、UAG、CAC、AAG、UGA、AGC、AGG |
|  | Amn | UGA、AGC、AGG、UAC、UAG、CAC、AAG、UCG、CCC、GUG |
| *rbcL* | Ae | CUC、UAG、UGA、CGG、AGG |
|  | Ai | UGA、CGG、UAG、ACG、AUA、CUC |
|  | Al | UGA、CGG、UAG、ACG、AUA、CUC |
|  | Aml | CUC、ACG、UAG、UGA、CGG |
|  | Amn | UGA、CGG、UAG、ACG、CUC、AUA |
| *rpl14* | Ae | CUC、UCU、UCG、CCU、CCG、ACA、ACG、GCC、UAC、UAG、CAC、CAG、UGC、UGA、UGG、CGC、AGG、GGC、GGG |
|  | Ai | CUC、UGC、UGA、UGG、AGG、GGC、UAC、UAG、CAC、UCU、UCC、UCG、CCU、CCG、ACA、GCC |
|  | Al | CUC、UGC、UGA、UGG、AGG、GGC、UAC、UAG、CAC、UCU、UCC、UCG、CCU、CCG、ACA、GCC |
|  | Aml | CUC、GUC、UCU、UCC、UCG、CCU、CCG、ACA、GCC、UAC、UAG、CAC、UGC、UGA、UGG、CGC、CGG、AGG、GGC |
|  | Amn | UGC、UGA、UGG、AGG、GGC、UAC、UAG、CAC、UCU、UCC、UCG、CCU、CCG、ACA、GCC、CUC |
| *rpl16* | Ae | UUG、CUC、CUG、GUC、GUG、UCA、CCG、ACG、GCC、UAC、UAG、CAC、AAC、GAU、UGC、UGA、CGG、AGC、GGC |
|  | Ai | UGC、UGA、CGG、AGC、GGC、UAG、CAC、AAC、GAU、UCA、CCG、ACG、GCC、UUG、CUC、CUG、GUG |
|  | Al | UGC、UGA、CGG、AGC、GGC、UAG、CAC、AAC、GAU、UCA、CCG、ACG、GCC、UUG、CUC、CUG、GUG |
|  | Aml | UUG、CUC、CUG、GUG、UCA、CCG、ACG、GCC、UAC、UAG、CAC、AAC、GAU、UGU、UGC、UGA、AGC、GGC |
|  | Amn | UGC、UGA、CGG、AGC、GGC、UAG、CAC、AAC、GAU、UCA、CCG、ACG、GCC、UUG、CUC、CUG、GUG |
| *rpl2* | Ae | UUC、UAA、UGC、UGA |
|  | Ai | UGA、UAA、UUC |
|  | Al | UGA、UAA、UUC |
|  | Aml | UUC、CUG、UAA、UGA |
|  | Amn | UGA、UAA、UUC |
| *rpl20* | Ae | UUC、CUC、GUG、CCU、CCC、CCA、CCG、ACG、GCG、UAG、CAC、GAC、GAG、UGC、UGA、CGC、GGU、GGC |
|  | Ai | UGC、UGA、AGG、GGU、GGC、UAG、CAC、GAC、GAG、CCU、CCC、CCA、CCG、ACG、UUC、CUC、GUG |
|  | Al | UGC、UGA、AGG、GGU、GGC、UAG、CAC、GAC、GAG、CCU、CCC、CCA、CCG、ACG、UUC、CUC、GUG |
|  | Aml | UUC、CUC、GUG、CCU、CCC、CCA、CCG、ACG、GCG、UAG、CAC、GAC、GAG、UGC、UGA、GGU、GGC |
|  | Amn | UGC、UGA、AGG、GGU、GGC、UAG、CAC、GAC、GAG、CCU、CCC、CCA、CCG、ACG、UUC、CUC、GUG |
| *rpl22* | Ae | CUG、GUC、UCG、CCA、CCG、ACG、GCC、UAG、CAC、CAG、UGA、AGG |
|  | Ai | UGA、CGG、UAG、CAC、UCG、CCA、CCG、ACG、GCC、CUC、CUG、AUC、GUC |
|  | Al | UGA、CGG、UAG、CAC、UCG、CCA、CCG、ACG、GCC、CUC、CUG、AUC、GUC |
|  | Aml | UUU、CUG、AUC、GUC、UCG、CCA、CCG、ACG、GCC、UAG、CAC、CAG、UGA |
|  | Amn | UGA、CGG、UAG、CAC、UCG、CCA、CCG、ACG、GCC、CUC、CUG、AUC、GUC |
| *rpoA* | Ae | ACC、UAC、UAG、UGA |
|  | Ai | UGA、UAC、UAG、ACC |
|  | Al | UGA、UAC、UAG、ACC |
|  | Aml | ACC、UAC、UAG、UGA |
|  | Amn | UGA、UAC、UAG、ACC |
| *rpoB* | Ae | UAG、UGA |
|  | Ai | UGA、UAG |
|  | Al | UGA、UAG |
|  | Aml | UAG、UGA |
|  | Amn | UGA、UAG |
| *rpoC1* | Ae | UAA、UAG、UGA |
|  | Ai | UGA、UAA、UAA |
|  | Al | UGA、UAA、UAA |
|  | Aml | UAA、UGA |
|  | Amn | UGA、UAA |
| *rpoC2* | Ae | UAA、UGA |
|  | Ai | UGA、UAA |
|  | Al | UGA、UAA |
|  | Aml | UAA、UAG |
|  | Amn | UGA、UAA |
| *rps11* | Ae | UUG、CUU、CUG、AUC、UCU、ACC、UAU、UAC、UAG、CAC、CAG、AAC、GAG、UGC、UGA、UGC |
|  | Ai | UGC、UGA、UCU、UAU、UAC、UAA、CAC、CAG、AAC、GAG、UUG、CUU、CUG、AUC |
|  | Al | UGC、UGA、UCU、UAU、UAC、UAA、CAC、CAG、AAC、GAG、UUG、CUU、CUG、AUC |
|  | Aml | UUG、CUU、CUG、UCU、UAU、UAC、UAA、CAG、AAC、GAG、UGC、UGA、CGC、AGC |
|  | Amn | UGC、UGA、UAU、UAC、UAA、CAC、CAG、AAC、UCU、UUG、CUU、CUG、AUC |
| *rps12* | Ae | UUC、UUG、CUC、CUG、UCG、CCG、GCA、GCG、UAC、UAG、GAC、GAG、UGC、UGA、UGG、AGU、AGC |
|  | Ai | UGC、UGA、UGG、CGG、AGU、AGC、UAC、UAG、GAC、GAG、UCC、CCG、GCA、GCG、UUC、UUG、CUC、CUG |
|  | Al | UGC、UGA、UGG、CGG、AGU、AGC、UAC、UAG、GAC、GAG、UCC、CCG、GCA、GCG、UUC、UUG、CUC、CUG |
|  | Aml | UUC、UUG、CUC、CUG、UCC、CCG、GCA、GCG、UAC、UAG、GAC、GAG、UGC、UGA、UGG、CGG、AGU、AGC |
|  | Amn | UGC、UGA、UGG、CGG、AGU、AGC、UAC、UAG、GAC、GAG、UCC、CCG、GCA、GCG、UUC、UUG、CUC、CUG |
| *rps14* | Ae | UUC、CUC、CUG、AUC、GUC、GUA、GUG、UCU、CCC、ACU、ACG、UAC、UAG、GAU、UGC、UGA、AGC、GGU、GGC、GGG |
|  | Ai | UGC、UGA、AGC、GGU、GGC、GGG、UAC、UAG、GAU、UCU、CCC、ACU、GCC、GCG、UUC、CUC、CUG、AUC、GUC、GUG |
|  | Al | UGC、UGA、AGC、GGU、GGC、GGG、UAC、UAG、GAU、UCU、CCC、ACU、GCC、GCG、UUC、CUC、CUG、AUC、GUC、GUG |
|  | Aml | UUC、CUC、CUG、AUC、GUC、GUA、GUG、UCU、CCC、ACU、GCC、GCG、UAC、UAG、GAU、UGC、UGA、AGC、GGU、GGC、GGG |
|  | Amn | UGC、UGA、AGC、GGU、GGC、GGG、UAC、UAG、GAU、UCU、CCC、ACU、GCC、GCG、UUC、CUC、CUG、AUC、GUC、GUG |
| *rps2* | Ae | GUC、UAC、UAA、UAG、CAC |
|  | Ai | UAC、UAA、UAG、CUA、GUC |
|  | Al | UAC、UAA、UAG、CUA、GUC |
|  | Aml | CUA、GUC、UCG、ACC、UAC、UAA、UAG |
|  | Amn | UAC、UAA、UAG、CUA、GUC |
| *rps3* | Ae | UUG、CUC、CUG、UCG、CCG、ACG、UAC、UAG、UGC、UGA、CGC、CGG、AGG |
|  | Ai | UGC、UGA、CGC、CGG、UAG、CAC、UCG、ACG、CUG |
|  | Al | UGC、UGA、CGC、CGG、UAG、CAC、UCG、ACG、CUG |
|  | Aml | CUC、GUG、UCG、ACG、UAG、CAC、UGC、UGA、CGC |
|  | Amn | UGC、UGA、CGC、CGG、UAG、CAC、UCG、ACG、CUG |
| *rps4* | Ae | CCG、ACG、UAG、UGU、UGA、AGC、GGC |
|  | Ai | UGU、UGA、CGG、AGC、GGC、UAG、CCG、CUC |
|  | Al | UGU、UGA、CGG、AGC、GGC、UAG、CCG、CUC |
|  | Aml | CUC、CCG、UAG、UGU、UGA、CGG、AGC、GGC |
|  | Amn | UGU、UGA、CGG、AGC、GGC、UAG、CCG、CUC |
| *rps7* | Ae | GUC、CUC、GUC、CCU、ACC、UAC、UAG、CAG、GAC、UGU、UGC、UGA、CGG、AGC、AGG |
|  | Ai | CCU、ACC、CUC、GUC、UGU、UGC、UGA、CGG、AGC、AGG、UAC、UAG、CAG、GAC |
|  | Al | CCU、ACC、CUC、GUC、UGU、UGC、UGA、CGG、AGC、AGG、UAC、UAG、CAG、GAC |
|  | Aml | CUC、GUC、CCU、ACC、UAC、UAG、CAG、GAC、UGU、UGC、UGA、CGG、AGC、AGG |
|  | Amn | UGU、UGC、UGA、CGG、AGC、AGG、UAC、UAG、CAG、GAC、CCU、ACC、CUC、GUC |
| *rps8* | Ae | CUC、GUA、GUG、UCA、UCG、CCG、ACG、GCC、GCA、GCG、UAC、UAG、CAC、CAG、AAG、UGC、UGA、CGU、AGC |
|  | Ai | UGC、UGA、CGU、AGC、UAC、UAG、CAC、CAG、AAG、UCA、UCG、CCA、CCG、ACG、GCC、GCG、CUC、GUA |
|  | Al | UGC、UGA、CGU、AGC、UAC、UAG、CAC、CAG、AAG、UCA、UCG、CCA、CCG、ACG、GCC、GCG、CUC、GUA |
|  | Aml | UUG、CUC、GUA、UCA、UCG、CCA、CCG、ACG、GCC、GCG、UAC、UAG、CAC、CAG、AAG、UGC、UGA、CGU、AGC |
|  | Amn | UGC、UGA、CGU、AGC、UAC、UAG、CAC、CAG、AAG、UCA、UCG、CCA、CCG、ACG、GCC、GCG、CUC、GUA |
| *ycf1* | Ae | UAG、UGA |
|  | Ai | UGA、UAG |
|  | Al | UGA、UAG |
|  | Aml | UAG、UGA |
|  | Amn | UGA、UAG |
| *ycf2* | Ae | UAG、UGA |
|  | Ai | UGA、UAG |
|  | Al | UGA、UAG |
|  | Aml | UAG、UGA |
|  | Amn | UGA、UAG |

Note: AI, *A. ilicifolius*; AE, *A. ebracteoctus*; AL, *A. leucostachyus*; AMON, *A. montanus*; AMOL, *A. mollis*; BP, *Barleria prionitis*; BC, *Blepharis ciliaris*; AP, *Aphelandra knappia*
